# Comparison and improvement of the predictability and interpretability with ensemble learning models in QSPR applications

**DOI:** 10.1186/s13321-020-0417-9

**Published:** 2020-03-30

**Authors:** Chia-Hsiu Chen, Kenichi Tanaka, Masaaki Kotera, Kimito Funatsu

**Affiliations:** Department of Chemical System Engineering, The University of Tokyo, 7-3-1 Hongo, Bunkyo-ku, Tokyo, 113-8656 Japan

**Keywords:** QSPR, Quantitative structure–property, Fluorescence, Liquid crystal, Ensemble learning, Blending, Decision tree, Random forest, Extremely randomized trees

## Abstract

Ensemble learning helps improve machine learning results by combining several models and allows the production of better predictive performance compared to a single model. It also benefits and accelerates the researches in quantitative structure–activity relationship (QSAR) and quantitative structure–property relationship (QSPR). With the growing number of ensemble learning models such as random forest, the effectiveness of QSAR/QSPR will be limited by the machine’s inability to interpret the predictions to researchers. In fact, many implementations of ensemble learning models are able to quantify the overall magnitude of each feature. For example, feature importance allows us to assess the relative importance of features and to interpret the predictions. However, different ensemble learning methods or implementations may lead to different feature selections for interpretation. In this paper, we compared the predictability and interpretability of four typical well-established ensemble learning models (Random forest, extreme randomized trees, adaptive boosting and gradient boosting) for regression and binary classification modeling tasks. Then, the blending methods were built by summarizing four different ensemble learning methods. The blending method led to better performance and a unification interpretation by summarizing individual predictions from different learning models. The important features of two case studies which gave us some valuable information to compound properties were discussed in detail in this report. QSPR modeling with interpretable machine learning techniques can move the chemical design forward to work more efficiently, confirm hypothesis and establish knowledge for better results.

## Introduction

Machine learning has led to an explosion of applications, and researchers have developed new capabilities of machine learning for a wide variety of tasks. There is growing interest in applications of machine-learning techniques in quantitative structure–activity relationship (QSAR) and quantitative structure–property relationship (QSPR) modeling research [1]. QSARs/QSPRs are models where characteristics of molecules are correlated with their experimental behaviors using various mathematical regression and classification algorithms [2].

One of the issues of QSAR/QSPR models is that they are difficult to interpret in a chemically meaningful manner. The effectiveness of QSPR will be limited by the machine’s inability to explain its thoughts and actions to researchers. While interpretable models can be easy to interpret simple such as linear regression [3] and decision trees [4], the most powerful algorithms with high accuracy like neural networks [5], support vector machine [6] are uninterpretable models, which provide predictions that are not designed to be interpretable and cannot be easily interpreted. Interpretable machine-learning approaches will be essential for researchers to understand, trust, and effectively manage. Approaches for interpreting a model can help to extract information from a model to justify its prediction. Moreover, the interpretation of the machine-learning model should be simple enough to be human-understandable. Thus, interpretable approaches should utilize interpretable models and interpretable descriptors. Interpretable descriptors must have clear structural or chemical meaning.

Traditional interpretable models such as linear regression or decision tree have unfavorable performance that we cannot merely squeeze much more accuracy out of any single model. New tools are being developed to create better interpretable models [7, 8]. One potential way to increase accuracy without losing too much interpretability is to combine the predictions of numbers of traditional interpretable models. It is called “ensemble learning.” Random forests (RF) is one of the examples of decision tree (DT) based ensemble learning models [9]. RF is typically treated as an uninterpretable model due to the complicated algorithm design. In fact, considering that the output of random forests is the majority vote by a large number of independent decision trees, and each tree is naturally interpretable. One efficient way to get an insight into a RF model is to compute feature importance [10]. In RF, it is not very hard to gauge the influence of individual features in a single tree at a time, but the global feature importance of RF can be quantified by the total decrease in node impurity averaged over all trees of the ensemble.

In QSAR/QSPR models, feature importance can be used to estimate the importance of single descriptors or group of descriptors representing different chemical properties to explain the relationships [11, 12]. Guha and Jurs [13] demonstrated that the RF model selected important descriptors similar to the multilinear regression and partial least square regression models. Polishchuk et al. [14] modeled the toxicity of 664 compounds toward tetrahymena pyriformis with RF and determined the importance of hydrophobic factors for toxicity variation. Marchese Robinson et al. [15] investigated different interpretation strategies for RF, linear SVM, and PLS models on several benchmark datasets, and the predictions were interpreted in a chemically and biologically meaningful way.

A benefit of using a DT-based ensemble learning models such as RF is that they can automatically provide estimates of feature importance from a trained predictive model. Generally, feature importance is a score that indicates how useful the feature was in the construction of the DTs within the model. However, the importance calculations are strongly based on the ensemble methods (such as bagging [16] or boosting [17]). For example, the learning in RF is done in parallel using “bagging,” and each tree is built from the random selection of features. On the other hands, the learning by “boosting” is done serially. Boosting tends to choose highly correlated features and use them in several trees. Therefore, different ensemble learning models may lead to different prediction results and different feature selections for interpretation. Therefore, the generalization of predictability and interpretability may be limited using feature importance provided by one specific DT-based ensemble learning model.

To solve this problem of different importance calculations, herein, we tried to ensemble the predictions of different DT-based ensemble learning models. The predictions from different models can simply be averaged, weighted, or combined in more mathematically sophisticated ways such as combinatorial QSAR modeling [18]. Super-learner (or meta-learner) is one of the great options as a more rigorous way to combine model predictions. The super-learner is a specific implementation of stacked generalization developed by Wolpert [19]. Stacked generalization uses a combiner model to decide the weights for the constituent predictions. “Blending” is very close to stacked generalization and can successfully improve prediction accuracy [20], but slightly more straightforward and less risk of an information leak than stacked generalization. In blending, the combining mechanism is that predictions from different models are used as training data for the super-learner (blender) to approximate the same target value. Basically, the blender can figure out the combining mechanism and do not affect the interpretability of each individual constituent model. Therefore, blending enhances understanding and leads to greater awareness and familiarity in a dataset by combining interpretable models.

In this paper, we proposed the development of a method for interpretable models based on “blending” to overcome the shortcomings of DT-based ensemble learning models. We used different blending methods to combine four different DT-based ensemble learning models for regression and binary classification modeling tasks. To validate the proposed method, we conducted two proof-of-concept case studies and confirmed that our method could propose preferable property values and interpretability. The established QSPR model for a regression task of fluorescence dataset was performed to study the fluorescence emission wavelengths of 413 fluorescent dyes in different solvent conditions. For the classification task, we used an organic compounds database with 3786 records to predict the liquid crystal behavior. We compared the predictive performances and important features from the blending method and four different DT-based ensemble models in both case studies. The important features used in these QSPR models which may give us some valuable information to properties were discussed in detail in this report. This study may lead to a better understanding of DT-based ensemble learning models and provide a meaningful manner for predictability and interpretability improvement.

## Method

### Dataset

For the two proof-of-concept studies, two different datasets were used: compounds for fluorescence dyes and liquid crystals.

#### 1. Fluorescence dataset

A large set of 413 dyes maximum experimental fluorescence wavelength (*λ*_em_) were collected in the database [21] and from several fluorescence researches [22–24]. The fluorescence dataset included a large variety of chromophore derivatives listed in Additional file 1: Table S1 such as cyanine, xanthene, coumarin, pyrene, naphthalene, anthracene, etc. A dataset containing 413 dyes, 473 samples for 418 dyes in different solvent conditions were used in this study. The data set was randomly divided into two subsets from each chromophore derivatives: a training dataset of 392 samples and a test dataset of 81 samples were used. The training set was used to evaluate their predictability of QSPR models.

#### 2. Liquid crystal dataset

A liquid crystal dataset was taken from LiqCryst database [25]. The dataset consisted of 3786 rod-like aromatic compounds with a variety of different mesogen types and wing substituents. There were 2780 liquid crystal (LC) compounds and 1006 compounds which LC behavior was not observed (NLC). For the purpose of developing the model, the dataset was randomly divided into the training set and test set, in the ratio 3:1. The training dataset consisted of 2060 LCs and 779 NLCs. The test dataset, which included 720 LCs and 227 NLCs, was used to test the developed models and to evaluate their generalization ability of classification.

### Model

#### Decision tree and ensemble learning

Decision tree (DT) is a popular method and shows many advantages over other simple models such as the classification and regression tree (CART) [4]. DT is very fast in training and requires practically no data preparation such as normalization or feature selection. Furthermore, DT is simple to understand and interpret using a flowchart-like structure. However, DTs have extremely low bias because they maximally overfit to the training data. Thus DTs are known to be unstable because small variations in the training data can result in different trees and different predictions. To address these shortcomings, ensemble learning algorithms have been proposed.

Ensemble learning algorithms are designed to improve the stability and accuracy of machine learning algorithms used in both classification and regression because they can be more accurate and robust than a single classifier or regressor [26–28]. The ensemble learning is that attempts to create a strong classifier/regressor from a number of weak classifiers/regressors. Fast and simple algorithms such as DT are commonly used as weak classifiers/regressors in ensemble learning methods. Several strategies are using in ensemble learning, such as bagging and boosting.

Bagging (stands for bootstrap aggregation) is a parallel ensemble method and aim to decrease the variance [16]. Bagging produces several subsets for training from the original dataset by random sampling with replacements, and each model is built independently. Bagging uses multiple models with high variance but low bias to obtain better predictions. For example, RF is one of the most popular and most powerful applications of bagging.

Boosting is a sequential ensemble method to decrease bias instead of variance [17]. It is a two-step approach, where boosting first uses subsets of the original data to build a model and then adds new models to reduce the error of previous models. Unlike bagging, every new subset contains the elements that were likely to be misclassified by previous models. The prediction of boosting is combined with those models using a particular cost function. Boosting uses multiple models with low variance but high bias models to obtain better predictions. AdaBoost is one of the best algorithms used to boost the performance of decision.

#### Random forests (RF)

Random forests (RF) is a versatile ensemble learning model using single full-grown DTs for both classification and regression tasks developed by Breiman [9]. Two types of randomness, bootstrap sampling and random selection of input variables, are used in the algorithm to make sure that the single DT grown in the forest are dissimilar and uncorrelated from each other. At each node of DT, the optimal split is derived by the reduction in impurity as CART. Growing a forest of trees with randomness leads to better predictions compared to a single DT and helps to make the model robust to noise in the data set.

#### Extremely randomized trees (ExtraTrees)

The ExtraTrees method is another ensemble learning model based on bagging which was introduced by Geurts et al. [29]. ExtraTrees is trained using bootstrap sampling and the random variable selection, like in an ordinary RF. But, the optimal cut-point at each node of DT during training is randomized. Subsequently, ExtraTrees is faster than RF when training. Geurts et al. established that ExtraTrees lead to a further decrease in overall variance. Furthermore, they have compared ExtraTrees with RF and shown to perform equal to or better than RF.

#### Adaptive boosting (AdaBoost)

AdaBoost is the first successful boosting algorithm developed by Freund and Schapire [17]. AdaBoost creates numbers of weak learners by adaptively adjusting the weights of each weak learner. After training a weak learner, AdaBoost increases the weight on the misclassified samples so that these samples will make up a larger part of the next weak learner training set. Then, the predictions of AdaBoost are made by majority vote of the weak learners’ predictions. Therefore, AdaBoost can generate expanding diversity to improve performance.

#### Gradient boosting (GBM)

Gradient boosting is another boosting algorithm similar to AdaBoost. The idea of gradient boosting is an optimization on a suitable cost function originated by Breiman [30]. This idea was further developed by Friedman [31, 32] and called gradient boosting machines (GBM). GBM also works by sequentially adding weak learners to an ensemble like AdaBoost. However, instead of tweaking the instance weights at every iteration as AdaBoost does, GBM tries to fit the new weak learner according to residual errors made by the previous weak learner. In other words, the algorithm of GBM is a numerical optimization problem to minimize the loss of weak learners using gradient descent. GBM has led to the development of boosting algorithms in many areas of machine learning.

### Feature importance

One efficient way of getting an insight into above DT-based ensemble models is to compute feature importance which is relatively straightforward to retrieve importance scores for each attribute. Feature importance can be calculated for a single DT by the amount that each attribute improves the performance measure. The performance measure is computing the amount of “impurity” such as variance in case of regression trees and Gini coefficient or entropy in case of classification trees. Generally, DT-based ensemble models provide a score that indicates how useful or valuable each feature was in the construction of the DTs within the model. The more an attribute is used to make key decisions with DTs, the higher its relative importance score. In other words, features with high importance scores are only important for the main split in DTs. The absolute value of the importance score is not as important as the relative values, which we can use to determine the most relevant features for a task. The basic use of the feature importance is to create a “feature ranking” among the features from high to low for each model.

### Blending

Ensemble learning is a procedure designed to increase predictive performance by combining the predictions of multiple machine learning models. There is a variety of ensemble methods, from simple ones like voting or averaging the predictions, to building complex learning models using the predictions as features. Stacked generalization is a way of combining predictions of multiple base models that have been demonstrated for a classification task [19], which has also been used for regression [33]. Stacked generalization applies a higher-level learning algorithm (so-called “meta-learner” or “super-learner”) and out-of-fold predictions for the training data to discover the best way of how to combine the outputs of the base models. Blending is very close to stacked generalization. Instead of creating out-of-fold predictions, blending is more straightforward and less risk of an information leak than stacked generalization.

**Fig. 1:**
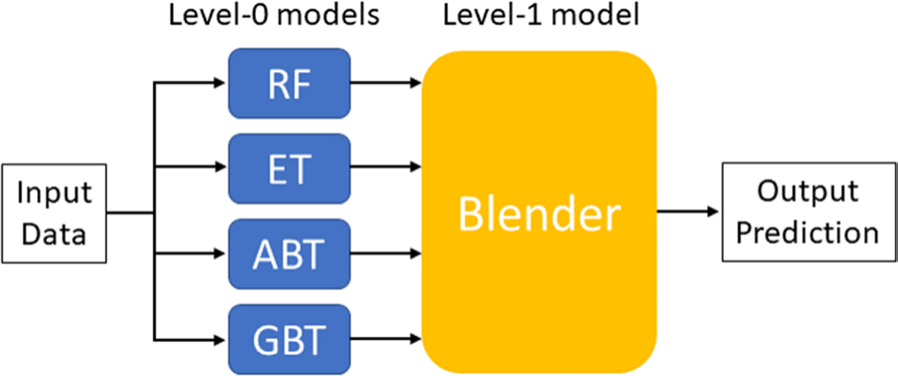
Concept of blending

There are two kinds of models in blending: several base models (level-0 models) and one blender (level-1 model) shown in Fig. 1. When using the blending for predictions, the training data is first fed into the level-0 models, and each of the level-0 model calculates a prediction value. These values are fed into the level-1 model; the level-1 model combines them and computes the final prediction. In other words, the inputs to the level-1 model are the outcomes of the level-0 models. Thus, the blender (level-1 model) decides if it wants to keep that level-0 model or not and summarizes information from level-0 models. The feature importance of blending was the summarization of level-0 model with different weights as following:

$$FI_{blend} = \mathop \sum \limits_{i = 1}^{n} w_{i} FI_{i}$$

where *FI*_blend_ is the feature importance of proposed method, *n* is the number of level-0 models, *w*_i_ is the weight of each level-0 model, and *FI*_i_ is the feature importance of each level-0 model. To ensure the predictability and interpretability preserved from each level-0 models, we used the lowest possible number of individual constituent models; then, we used simple voting, linear combinations, or DT-based ensemble learning as a level-1 model. In this work, we use three different blenders to combine results of level-0 models. To simply compare the different performance of blenders, we defined three blending methods as following:

- Uniform blending: use simple voting in classification and average in regression as blenders. The weights (*w*_i_) of each model were the same.
- Linear blending: use a linear model as a blender.
  We used logistic regression in a classification task and multiple linear regression in a regression task. The weights (*w*_i_) of each model were determined by regression coefficients.
- Any blending: use a non-linear model as a blender.
- We used GBM with simple structures (n_estimators = 10) as the level-1 model because of the serial dependence of level-0 models. The weights (*w*_i_) of each model were determined by the feature importance of GBM.

### Descriptor selection

Many studies have noted to achieve model interpretation in many research fields in QSAR/QSPR using interpretable descriptors [34, 35]. Therefore, we used different interpretable descriptor sets for two case studies.

Dragon 7 software [36], Gaussian 09 software [37] and RDKit package [38] were used for descriptor calculation. In the case study of fluorescence dataset, 2143 Dragon 7 molecular descriptors from 0-dimensional to 2-dimensional molecular information and 25 quantum chemical (QC) descriptors were calculated by Gaussian 09 software. The geometries of the molecules were optimized with the B3LYP density functional method [39], 6–31G* basis set, and frequency calculations. For improving the prediction accuracy, Dragon 7 descriptors demonstrated better prediction than RDKit that the result is in Additional file 1: Table S2. 3-dimensional Dragon 7 molecular descriptors were not considered since they are hardly interpreted. The use of QC descriptors can successfully improve prediction and interpretability from our previous work [40] and provide more specific physical meanings. We consider solvent species in model constructing since the solvent effect plays a more critical role in the process of fluorescence [41].

**Fig. 2:**
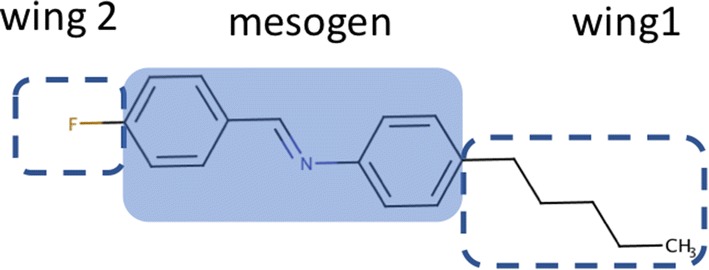
Structure template of rod-like LC

In the case study of liquid crystal dataset, RDKit descriptors were applied in this research for improving the accuracy of LC behavior prediction because Dragon 7 descriptors did not improve predictability. Our previous works noted that structural descriptors resulted in good LC prediction [42], we only calculated interpretable structural descriptors in RDKit such as atom counts and numbers of fragments. The comparison of Dragon 7 and RDKit descriptors is listed in Additional file 1. Furthermore, we separated the structural template of LC into mesogens and wings for descriptor calculations to improve interpretability shown in Fig. 2. Total 250 interpretable descriptors were calculated by RDKits containing 84 descriptors from a raw structure, 72 descriptors from mesogen, 46 descriptors from wing1 and 48 descriptors from wing2. The detail of descriptor selection is provided in Additional file 1.

### Evaluation of model performance

The performance of created regression models was evaluated by coefficient of determination (*R*^2^) and root mean square error (RMSE).

$${\text{RMSE}} = \sqrt {\frac{1}{n}\mathop \sum \limits_{j = 1}^{n} \left( {y_{j} - \widehat{y}_{j} } \right)^{2} }$$

where $y_{j}$ is the observed value for the *j*th observation, $\widehat{y}_{j}$ is the predicted value and n is the number of samples.

The performance of created classification models was analyzed on the basis of classification results obtained for the prediction set. The used performance metrics are defined as follows:

Accuracy $$Acc = \frac{a + d}{a + b + c + d}$$

Precision $$\Pr = \frac{a}{a + c}$$

Recall $$r = \frac{a}{a + b}$$

F1 score $$F1 = \frac{2}{{\frac{1}{\Pr } + \frac{1}{r}}} = \frac{2a}{2a + b + c}$$

Matthews correlation coefficient (*MCC*)

$${\text{MCC}} = \frac{a \times d - b \times c}{{\sqrt {\left( {a + b} \right)\left( {a + c} \right)\left( {b + d} \right)\left( {c + d} \right)} }}$$

where *a* is true positive, *b* is false negative, *c* is false positive, and *d* is true negative (Table 1).

**Table 1:** Confusion table

Actual class	Predicted class	
	LC	NLC
LC	*a*	*b*
NLC	*c*	*d*

Accuracy (*Acc*) gives the percentage of LCs and NLCs correctly classified, while the precision (*Pr*) gives the percentage of correctly classified LCs among all compounds which are classified as LCs. The recall (*r*) represents the numerical value of the probability of identifying compounds that exhibit the LC phases. The F1 score (*F1*) can be interpreted as a weighted average of precision and recall. This score takes both false positives and false negatives into account. The Matthews correlation coefficient (*MCC*) is a correlation coefficient to handle imbalanced data between the observed and predicted binary classifications. It is not as easy to understand as accuracy intuitively, but the *F1* and *MCC* are usually more useful than accuracy, especially for imbalanced class distribution.

### Software and implementation

Four DT-based ensemble learning models are freely available in Python. RF, ExtraTrees, AdaBoost, and GBM were constructed using Scikit-learn package in Python [43]. All models are able to compute feature importance automatically for every feature after training. All descriptors in this study were calculated by Dragon 7 and RDKit. Statistical analyses were conducted using Python scripts.

## Results and discussion

### Case study 1: fluorescence dataset

#### Performance of DT-based ensemble models

To obtain DT-based ensemble learning models, the hyper-parameters were determined based on the root mean squared error (RMSE) of fivefold cross-validation using a randomized search. The overall performances for fluorescence wavelength (*λ*_em_) of four different DT-based ensemble learning approaches are presented in Table 2. Figures showing the predicted *λ*_em_ versus experimental *λ*_em_ for training dataset and test dataset are in Additional file 1: Figs. S3–S6).

**Table 2:** The results of four different DT-based ensemble learning methods

	Training dataset		Test dataset	
	$R^{2}$	RMSE (nm)	$R_{pred}^{2}$	RMSE (nm)
RF	0.966	22.25	0.904	34.42
ExtraTrees	0.991	11.15	0.908	33.71
AdaBoost	0.981	16.22	0.904	34.45
GBM	0.988	12.92	0.905	34.26

The performances of all of the four DT-based ensemble learning models were similar in agreement of our previous work results which ensemble learning was suitable for fluorescence prediction than single models [40]. The best well-fitted model was ExtraTrees with $R^{2}$ = 0.991, RMSE = 11.15 nm for training dataset which demonstrated good predictability for the external test dataset ($R_{pred}^{2}$ = 0.908, RMSE = 33.71 nm). In machine learning, there is something called the “No Free Lunch” theorem which states that no machine learning model can typically capture the full complexity of problems. Although all models demonstrated nearly the same results, each model only provides a rough representation of the problem to solve. Different models may be likely to have various prediction results. Therefore, the interpretation of models may help to understand the factors of model predictions.

#### Interpretation of DT-based ensemble models

The feature importance provided by DT-based ensemble learning models can help identify input variables that may be most relevant of each descriptor to the regression problems. Since the random state may influence the feature importance in ensemble learning models, every model was repeated ten times with different random states from 0 to 10. The basic use of the feature importance is to create a “feature ranking” among the features from high to low for each model. It is meaningless to compare the values of feature importance of different models. We hence discuss the top 10 important descriptors among 2169 descriptors in four models which were highly related to fluorescence wavelengths.

Table 3 shows that ten important descriptors and their feature importance from four DT-based ensemble learning models for the fluorescence wavelength. The feature importance of HOMO–LUMO gap is far greater than other descriptors. The rest descriptors had relatively lower scores of feature importance than the HOMO–LUMO gap. In our opinion, models made the main prediction decision based on HOMO–LUMO gap and used the rest descriptors for minor correlation in predictions because the emission process is the energy relaxation from LUMO to HOMO.

**Table 3:** Top 10 important descriptors selected by four DT-based ensemble learning models

RF		ExtraTrees	
Selected descriptors	Feature importance	Selected descriptors	Feature importance
Gap	0.3412	Gap	0.0712
AP(xx)	0.0986	F01[C-N]	0.0350
Chi1_EA(dm)	0.0344	AP(xx)	0.0243
Chi0_EA(dm)	0.0274	SpMax2_Bh(i)	0.0221
EP(xx)	0.0239	F02[C-N]	0.0192
P_VSA_ppp_L	0.0215	SpMax7_Bh(m)	0.0179
SpDiam_AEA(ed)	0.0160	F01[C-C]	0.0174
SpMax_AEA(ed)	0.0134	C-004	0.0157
SpMin5_Bh(m)	0.0119	P_VSA_e_2	0.0152
CATS2D_06_LL	0.0093	EP(xx)	0.0123

AdaBoost		GBM	
Selected descriptors	Feature importance	Selected descriptors	Feature importance
Gap	0.1196	Gap	0.1621
AP(xx)	0.0682	Solvent	0.0534
P_VSA_MR_7	0.0601	MATS1e	0.0147
SpMax2_Bh(i)	0.0408	Chi1_EA(dm)	0.0108
F01[C-N]	0.0382	MATS6m	0.0107
P_VSA_ppp_L	0.0317	SpMax_AEA(ed)	0.0102
F02[C-N]	0.0224	Eig01_AEA(ed)	0.0100
LUMO	0.0197	CATS2D_00_LL	0.0092
EP(xx)	0.0167	AP(xx)	0.0089
SdsCH	0.0141	SpMin8_Bh(e)	0.0086

In RF, the feature importance of HOMO–LUMO gap was three times larger than AP(xx) which HOMO–LUMO gap dominated the main predictions. RF used topological descriptors in minor correlations for final predictions. The feature importance scores of topological descriptors were much smaller than the HOMO–LUMO gap which means less effect to predictions. In ExtraTrees, F01[C-N] was as important as AP(xx) because of the large number of cyanine dyes in training dataset. Unlike RF, HOMO–LUMO gap did not have extremely high importance than other descriptors since the ExtraTrees did not optimize cut-points of DTs in ExtraTrees. ExtraTrees used more structural descriptors (F01[C-N], F01[C-N], F01[C-C] and C-004) which also resulted from cyanine dyes. In AdaBoost, more QC descriptors were selected to build DT weak learners since the depth of DTs in AdaBoost is only five. Similar to ExtraTrees, structural descriptors also had high importance scores in AdaBoost. In GBM, except the HOMO–LUMO gap and solvent, the rest descriptors had small importance scores like RF. The descriptors selected by GBM were apparently different from the other three models. Interestingly, GBM was the only model that focus on solvent effect.

Obviously, four DT-based ensemble learning models used different descriptors and prediction mechanisms to approach fluorescence prediction. There is no absolute answer which model is the correct model for prediction and interpretation because the fluorescence is a complex phenomenon. It is hard to interpret the relationship between descriptors and properties because the information from four different models is too scattered. One of the best ways to improve predictions and extract information from prediction models is to combine or summarize different models. Thus, we tried to use blending to summarize information from four different DT-base ensemble learning models and discuss the result in the next section.

#### Performance of blending models

The prediction results of four DT-base ensemble learning models (level-0 models) were fed into the blending model (level-1 models). Three different blending methods are listed as follows:

- Uniform blending: average the prediction results and feature importance (no meta-learning).
- Linear blending: use multiple linear regression to summarize information (linear meta-learning).
- Any blending: use GBM regression to summarize information (non-linear meta-learning).

**Fig. 3:**
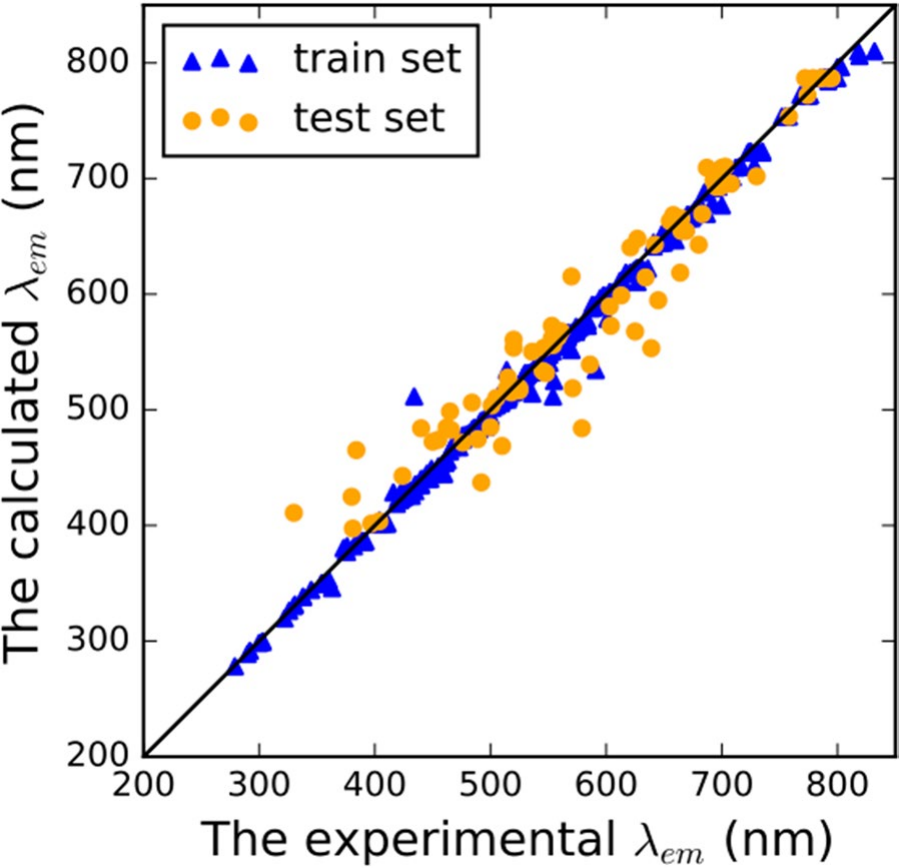
Experimental values versus calculated values of λ_em_ by any blending

The results of the blending models are shown in Table 4. The summarization of level-0 models with blending successfully improved the prediction performance of fluorescence prediction. Simple summarization of level-0 models such as uniform blending and linear blending can efficiently overcome the pros and cons of level-0 models. Any blending exhibited the best prediction and large improvement from level-0 models. The only difference is that there are same weights of level-0 models in uniform blending but different weights (RF:10%, ExtraTrees:56%, AdaBoost:20%, GBM:14%) in linear blending. Above three blending methods, any blending was performed to develop a nonlinear relationship between level-0 models and fluorescence wavelength. To obtain better results, the hyper-parameters (n_estimators = 10, max_depth = 8, learning_rate = 0.1) that influence the performance of level-1 GBM were optimized by grid search with fivefold cross-validation. The $R^{2}$ of the training dataset was 0.996, and the $R^{2}$ of the test dataset was 0.931. Any blending model had the RMSE of 7.84 nm for the training dataset, 29.11 nm for the test dataset. Figure 3 shows the experimental values versus calculated values of *λ*_em_ by any blending. The improvement of any blending was larger than uniform blending and linear blending. Therefore, it reveals that none of level-0 models had the best answer but the ensemble of level-0 models can lead to better predictions.

**Table 4:** The results of three different blending methods

	Training dataset		Test dataset	
	$R^{2}$	RMSE (nm)	$R_{pred}^{2}$	RMSE (nm)
Uniform blending	0.988	13.26	0.921	31.35
Linear blending	0.992	10.25	0.922	31.05
Any blending	0.996	7.84	0.931	29.11

**Fig. 4:**
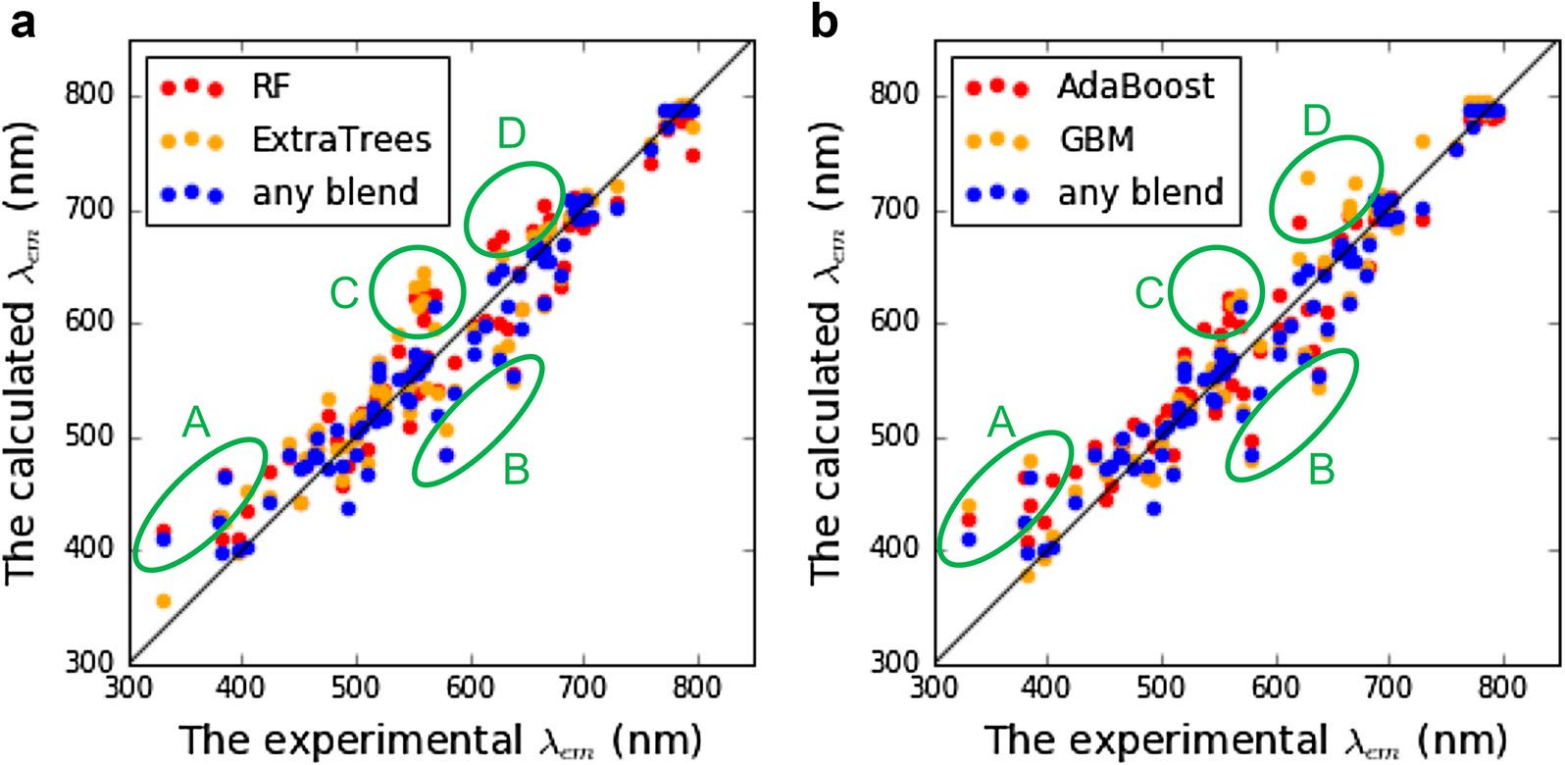
Performance comparison of **a** RF, ExtraTrees and any blending, **b** AdaBoost, GBM and any blending

**Fig. 5:**
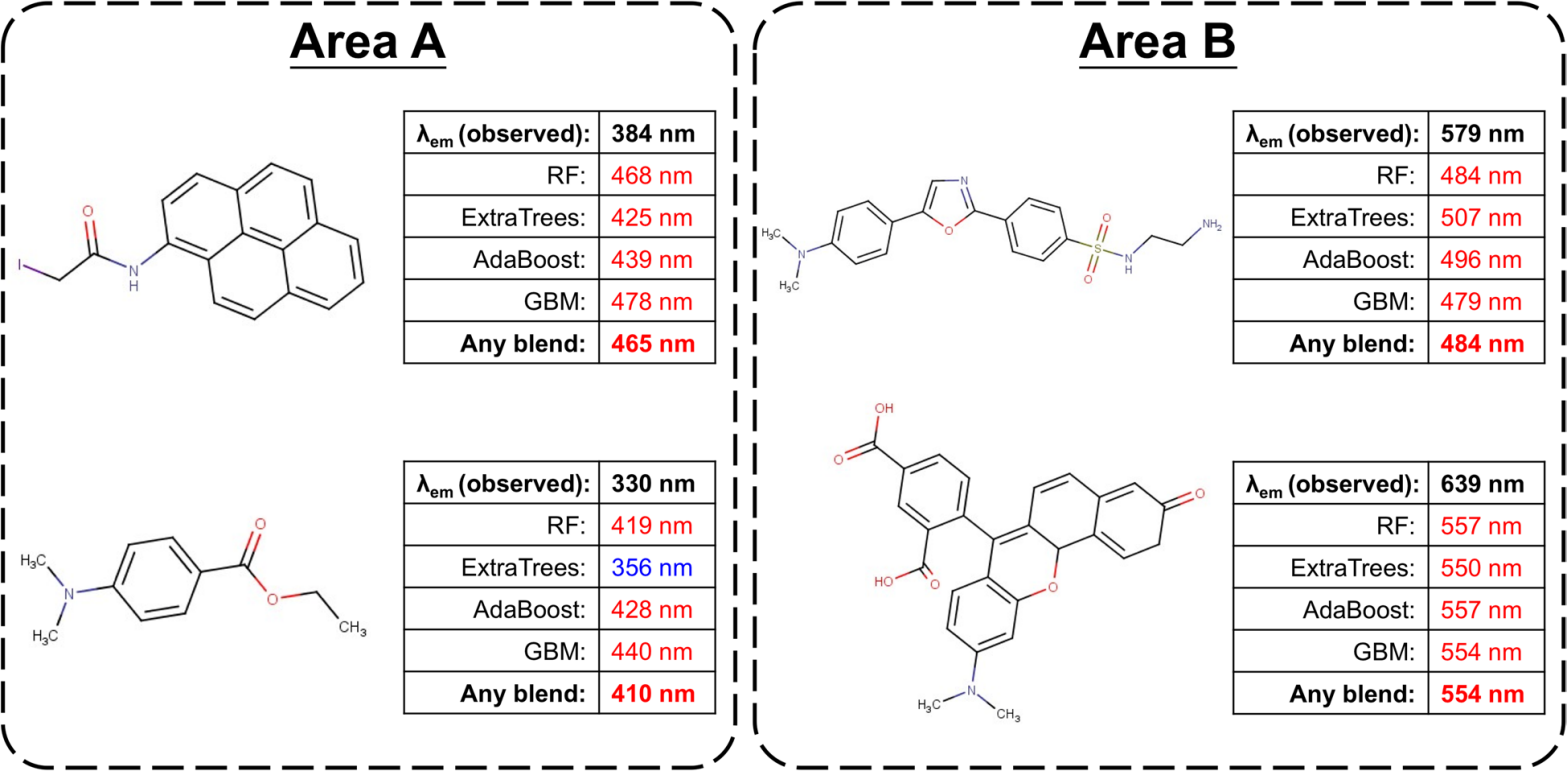
Chemical structures in area A and area B which every prediction models could not provide accurate predictions

**Fig. 6:**
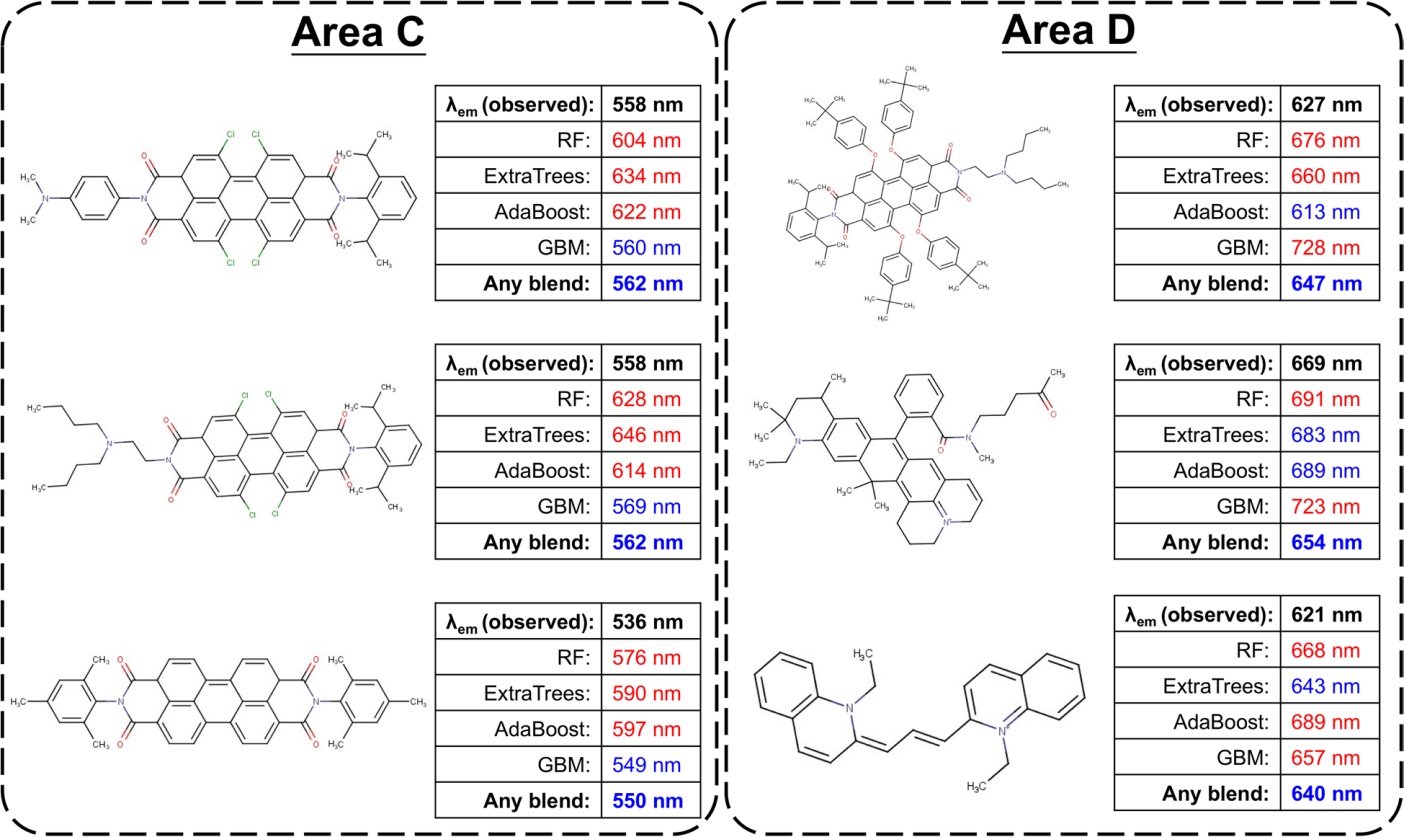
Chemical structures in area C and are D which specific models could not provide accurate predictions

Performance standards such as *R*^2^ and RMSE play crucial roles in determining the success or failure of model training and performance improvement efforts. However, the differences between models were not significant. In fact, blending only can provide small improvements since DT-based ensemble learning models are already powerful algorithms. Due to the complexity of fluorescence mechanisms, none of the models are perfect. Thus, we not only compared the difference between standards but also examined the difference in predictions of test samples. Figure 4 reveals experimental *λ*_em_ values versus calculated *λ*_em_ values of the test set using RF, ExtraTrees, AdaBoost, GBM and any blending. In Fig. 4, we highlighted four different areas with large prediction error in some models, and some chemical structure examples are listed in Figs. 5, 6.

Every model failed to predict the *λ*_em_ of structures in area A and area B because four ensemble learning models cannot successfully capture the patterns of the fluorescence phenomena. In area A, any blending could slightly reduce the prediction error by using the accurate prediction provided by ExtraTrees. The two structures in area B had long fluorescence wavelengths due to the solvent effect, but lack of samples in different solvent causes the worst predictions.

Any blend performed better prediction in area C and area D among all ensemble learning models with better R^2^ and RMSE. In area C, the significant weakness of RF, ExtraTrees and AdaBoost was the prediction of tetracarboxylic dianhydride structures. The correct predictions of any blending only relied on predictions of GBM. On the other hand, three dyes in area D were hard to be predicted by RF and GBM. Any blending provided better predictions based on ExtraTrees and AdaBoost. In summary, four DT-based ensemble models had their own pros and cons for predictions of specific dyes. Therefore, the improvement provided by any blending is not only the performance standards but also summarization of different models.

#### Interpretation of blending models

Table 5 shows that ten important descriptors and feature importance from three blending methods. Obviously, unlike the feature importance results of level-models, three blending methods selected similar descriptors but the score and ranking of each descriptor were slightly different. Despite the small difference caused by each blending method, the summarized information led to a unification explanation which was easier for interpretation of models.

**Table 5:** Top 10 important descriptors selected by three blending methods

Uniform blending		Linear blending		Any blending	
Selected descriptors	Feature importance	Selected descriptors	Feature importance	Selected descriptors	Feature importance
Gap	0.1654	Gap	0.1330	*Gap*	*0.1572*
AP(xx)	0.0470	AP(xx)	0.0359	*AP(xx)*	*0.0411*
F01[C-N]	0.0216	F01[C-N]	0.0247	*Solvent*	*0.0224*
SpMax2_Bh(i)	0.0176	SpMax2_Bh(i)	0.0174	F01[C-N]	0.0198
Solvent	0.0169	Solvent	0.0141	SpMax2_Bh(i)	0.0170
P_VSA_MR_7	0.0162	F02[C-N]	0.0133	P_VSA_ppp_L	0.0147
P_VSA_ppp_L	0.0151	EP(xx)	0.0122	EP(xx)	0.0115
EP(xx)	0.0133	F01[C-C]	0.0111	F02[C-N]	0.0108
Chi1_EA(dm)	0.0119	SpMin5_Bh(m)	0.0106	Chi1_EA(dm)	0.0105
F02[C-N]	0.0109	P_VSA_ppp_L	0.0103	Chi0_EA(dm)	0.0092

Italic values indicate the significance of important features which highly affect the fluorescence wavelengths

We discuss the top ten important descriptors in any blending models because of the high predictive accuracy for fluorescence wavelength. The ten important descriptors also contained three QC descriptors. The HOMO–LUMO gap, Van der Waals surface areas (P_VSA_ppp_L) can be explained as the absorption process of compounds. For example, dyes with large conjugation area such as cyanine dyes result to the large Van der Waals surface area. SpMax2_Bh(i), a topological descriptor based on ionization potential, may be related to the ionization in solvents that is able to change the spectral characteristics of the dye [44]. The fluorescence wavelength has strong solvent effects, as called “solvatochromism” based on the change of polarities of the ground and excited state of a chromophore in the solvent polarity [41]. The high importance of solvent species, polarizability (AP(xx), EP(xx)) and dipole moment correlated topology descriptors (Chi1_EA(dm), Chi0_EA(dm)) supported the fact of solvatochromism phenomena. The structural descriptors such as F01[C-N] and F02[C-N] reflected the structural features of cyanine dyes with the large ratio in training dataset.

### Case study 2: liquid crystal dataset

#### Performance of DT-based ensemble models

**Fig. 7:**
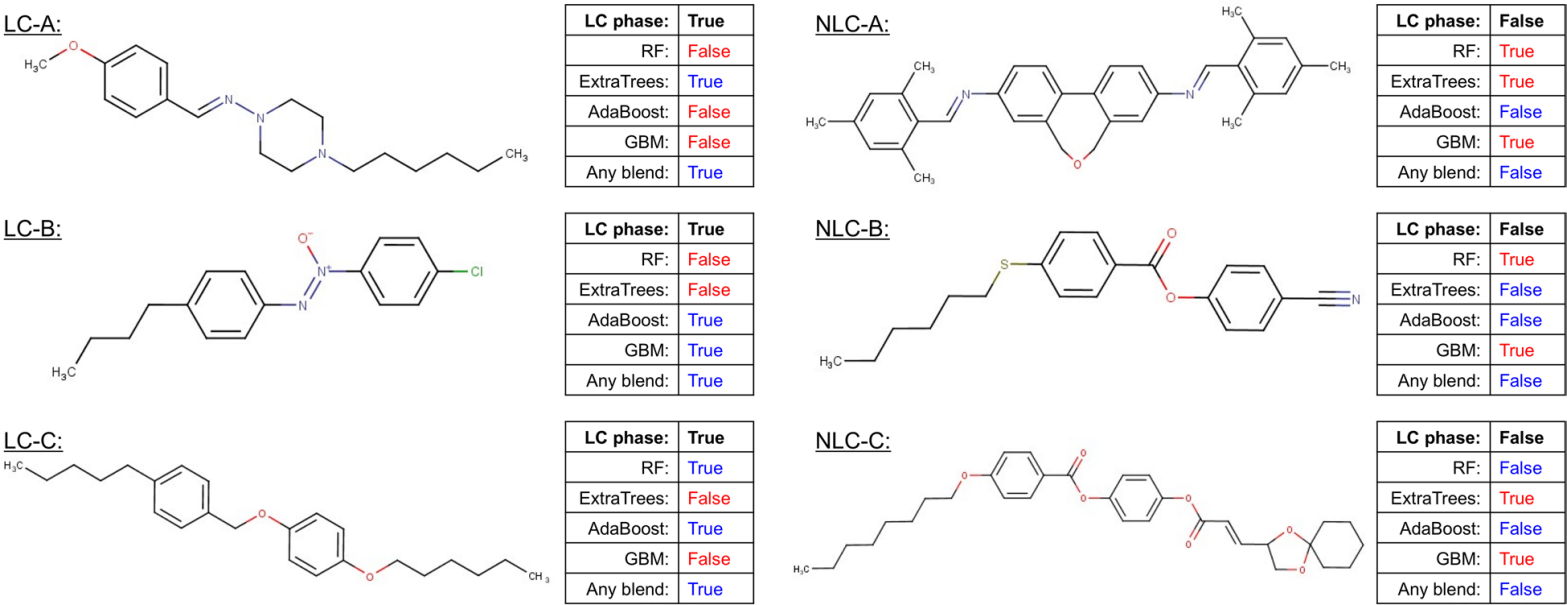
Prediction comparison of three LC compounds (LC-A, LC-B and LC-C) and three NLC compounds (NLC-A, NLC-B and NLC-C)

The optimized hyper-parameters are listed in Additional file 1 and were determined based on the accuracy of fivefold cross-validation using a randomized search. Like the results of the regression task, the classification results of liquid crystal dataset by different modeling approaches are similar. From the results of four different DT-based ensemble learning models in Table 6, RF had the best performance of LC prediction among four ensemble learning models with the highest *F1* and *MCC*. AdaBoost also had similar *F1* but smaller *MCC* due to the less balance of precision and recall. However, the differences in predictability among the four models were limited. Both high bias unpruned DT with bagging strategy (RF and ExtraTrees) and high variance DT with boosting strategy (AdaBoost and GBM) both reached the same goal to predict LC properties. However, different DT-based ensemble learning models provided different predictions on the same compound. In the test dataset, there were 102 compounds which four DT-based ensemble learning models could not offer consistent prediction results. Figure 7 illustrates some examples of these compounds with consistent prediction results. We will compare the single prediction result of different models later. Moreover, we will discover the insights of four DT-based models from feature importance of how they make predictions.

**Table 6:** Performance metrics values and corresponding confusion tables for four different classifiers

	Training set		Test set							
	*Acc* (%)	*F1* (%)	*Acc* (%)	*Pr* (%)	*r* (%)	*F1* (%)	*MCC* (%)	Actual class	Predicted class	
									LC	NLC
RF	99.3	99.5	88.5	91.7	93.5	92.5	67.3	LC	673	47
								NLC	61	166
ExtraTrees	99.3	99.5	87.5	91.2	92.5	91.9	65.2	LC	666	54
								NLC	64	163
AdaBoost	99.3	99.5	88.1	91.2	93.4	92.3	65.1	LC	673	47
								NLC	65	162
GBM	95.3	96.8	87.4	91.0	92.6	91.8	64.3	LC	667	53
								NLC	66	161

#### Interpretation of DT-based ensemble models

Table 7 demonstrates five important descriptors and feature importance from four DT-based ensemble learning models for the prediction of LC, and every model was repeated ten times with random states from 0 to 10. The detailed bar charts of feature importance containing 20 important descriptors are listed in Additional file 1. Unlike the results of fluorescence dyes that had only one descriptor with extremely high importance score, the importance scores of the top five important descriptors in LC prediction were gradual decrements. In other words, the descriptor rank of LC prediction is more meaningful than fluorescence prediction because the model did not rely on one specific descriptor for prediction. Thus, we selected the top five important descriptors to discuss the differences between the four models. RF and ExtraTrees chose similar descriptors toward prediction. Two wing descriptors, wing1_NumRotatableBonds, and fr_unbrch_alkane were within in top six in RF and ExtraTrees models shown in Additional file 1: Figs. S9, S10. HeavyAtomCount and NumRotatableBonds had almost the same importance in both models.

**Table 7:** Top five important descriptors of LC selected by four DT-based ensemble learning models

RF		ExtraTrees	
Selected descriptors	Feature importance	Selected descriptors	Feature importance
HeavyAtomCount	0.04649	NumRotatableBonds	0.03541
NumRotatableBonds	0.04381	HeavyAtomCount	0.03495
wing2_HeavyAtomCount	0.04329	wing1_NumRotatableBonds	0.02801
fr_unbrch_alkane	0.03315	wing2_HeavyAtomCount	0.02700
wing1_NumRotatableBonds	0.03218	wing1_HeavyAtomCount	0.02653

AdaBoost		GBM	
Selected descriptors	Feature importance	Selected descriptors	Feature importance
HeavyAtomCount	0.08812	HeavyAtomCount	0.07017
NumRotatableBonds	0.06759	mesogen_HeavyAtomCount	0.05329
wing2_HeavyAtomCount	0.05722	NumRotatableBonds	0.04532
wing1_HeavyAtomCount	0.04705	mesogen_NumRotatableBonds	0.02774
mesogen HeavyAtomCount	0.04462	wing2_HeavyAtomCount	0.02718

On the other hands, boosting models had different results of feature importance even though the performances of boosting models were the same as bagging models. GBM used more mesogen descriptors for prediction than AdaBoost; instead, RF and ExtraTrees used more wing descriptors. The importance scores of AdaBoost and GBM are relatively larger than RF and ExtraTrees because shallow DTs in AdaBoost GBM picked up fewer features in training than full-grown DTs in RF and ExtraTrees. Interestingly, important descriptors selected by GBM were largely different from other models. In GBM, HeavyAtomCount, mesogen_HeavyAtomCount, and NumRotatableBonds were dominant factors of LC prediction. In consequence, four models still had slightly different processes and scenarios in LC prediction. It would be better for improving the prediction and interpretation by summarizing four models using blending. The relationships between descriptors and LC properties will be discussed in the next section.

#### Performance of blending models

In LC prediction, three different blending methods are listed as follows:

- Uniform blending: vote the prediction results and average feature importance (no meta-learning).
- Linear blending: use logistic regression to summarize information (linear meta-learning).
- Any blending: use GBM classification to summarize information (non-linear meta-learning).

The blending results obtained for LC prediction are presented in Table 8. As a result, three blending methods only had slight improvements compared to 4 DT-based ensemble learning models (level-0 models). The confusion table reveals that linear blending and any blending increase the correct prediction of NLCs. Any blending with 10 estimators and max_depth = 4 as hyperparameters were performed to develop a better result between level-0 models and LC property, demonstrating a high accuracy of 88.8% and the F1 score of 92.7% and the MCC of 68.6%. Although it is hard to conclude that blending had significant improvements based on performance metrics, the advantage of blending is to summarize the different predictions of level-0 models. We further compared the prediction results of 4 DT-based ensemble learning models and any lending of three LC structures and three NLC structures. It is risky to rely on one specific model for predicting complex chemical phenomena. Thus, we further compared the prediction results of four DT-based ensemble learning models and any lending of three LC structures and three NLC structures shown in Fig. 7. These examples reveal that there were pros and cons using different models for the same prediction. To improve the generalization of predictability, the blending method was one of the solutions to summarize different prediction results. Surprisingly, any blending provided correct prediction from only one model with correct prediction such as LC-A in Fig. 7 and NLC-A in Fig. 7. Thus, blending methods solved the inconsistency of level-0 model predictions as long as over two level-models had correct predictions. Although, blending methods did not provide a significant improvement of prediction, the summarization of level-0 model information was the crucial application of blending. To further understand the detail and effect of blending, it is necessary to compare the feature importance of descriptors extracted by different blending methods.

**Table 8:** Performance metrics values and corresponding confusion tables for three different blending methods

	Training set		Test set							
	Acc (%)	F1 (%)	Acc (%)	Pr (%)	r (%)	F1 (%)	MCC (%)	Actual class	Predicted class	
									LC	NLC
Uniform blending	99.5	99.7	88.3	91.3	93.6	92.5	67.3	LC	674	46
								NLC	64	163
Linear blending	99.5	99.7	88.4	91.6	93.5	92.5	67.8	LC	673	47
								NLC	62	165
Any blending	99.3	99.5	88.8	91.7	93.8	92.7	68.6	LC	675	45
								NLC	61	166

#### Interpretation of blending models

We selected the top five important descriptors and feature importance from three blending methods in Table 9. The bar charts of feature importance are listed in Additional file 1. First, we compared the descriptor selections of different blending methods; then we discussed why those descriptors were important to LC prediction. Uniform blending and linear blending had almost same *Acc* and *F1*. They used similar descriptors in prediction (detailed in Additional file 1: Figs. S13, S14). Due to the equal weights of level-0 models in uniform blending, AdaBoost and GBM with larger importance scores led to the higher importance of mesogen descriptors. On the other hands, the weights of level-0 models were derived from coefficients of in logistic regression (RF:11%, ExtraTrees:47%, AdaBoost:11%, GBM:31%). Thus, the linear blending result was mainly based on ExtraTrees models so that the wing descriptors were more important than mesogen descriptors.

**Table 9:** Top five important descriptors selected by three blending methods

Uniform blending		Linear blending	
Selected descriptors	Feature importance	Selected descriptors	Feature importance
HeavyAtomCount	0.05993	HeavyAtomCount	0.04781
NumRotatableBonds	0.04803	NumRotatableBonds	0.04219
wing2_HeavyAtomCount	0.03827	wing2_HeavyAtomCount	0.03471
mesogen_HeavyAtomCount	0.03484	wing1_HeavyAtomCount	0.03006
wing1_HeavyAtomCount	0.03290	fr_unbrch_alkane	0.02897

Any blending			
Selected descriptors	Feature importance		
HeavyAtomCount	0.06520		
NumRotatableBonds	0.05236		
wing2_HeavyAtomCount	0.04321		
wing1_HeavyAtomCount	0.03636		
mesogen_HeavyAtomCount	0.03556		

Any blending used the same top five descriptors as uniform blending depended on weights (RF: 49%, ExtraTrees: 6%, AdaBoost: 8%, GBM: 37%) derived from feature importance of the level-1 GBM model. The high usage rate of GBM led to the higher importance of mesogen descriptors. There are two possible reasons why any blending improved the prediction performance. One reason is the high weight of RF since RF was the best prediction model among four level-0 models. The second reason is the ensemble learning of level-1 GBM model which can successfully solve the complicated relationship between predictions of level-1 models and LC behavior.

**Fig. 8:**
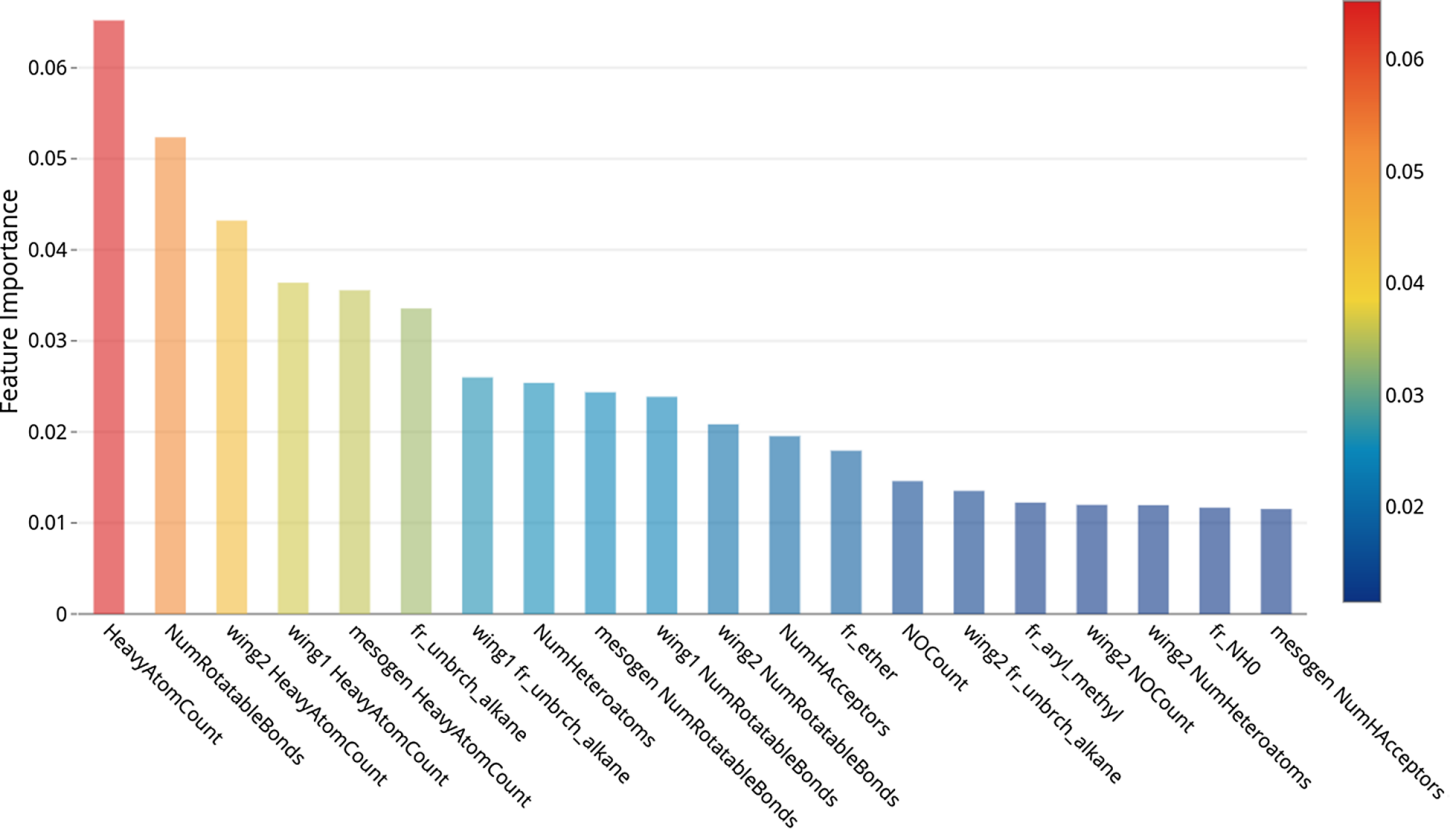
Bar chart of top 10 important descriptors selected by any blending

Lastly, the summarized information led to a unification interpretation of relationships between descriptors and LC properties. Two descriptors calculated from the raw structure, HeavyAtomCount and NumRotatableBonds, were almost selected by every model. HeavyAtomCount represents numbers of atoms except for hydrogens which can be regarded as the size of compounds, and NumRotatableBonds stands for alkyl chains. As a matter of fact, LCs consistent with long alkyl groups, and the molecular length should be at least 1.3 nm [45]. The rest important descriptors were the size of wing1, wing2, and mesogens. In our structure separation, we defined that wing1 is a longer chain and wing2 is a shorter one. The long hydrocarbon of wing1 such as alkyl chains is able to stabilize molecular orientations to form liquid crystal phases [46]. Some wing2 fragments are small polar groups such as halogens, nitrile groups, and nitro groups which also generate intermolecular force to stabilize orientations [46]. The importance score of mesogen size was high because an extended, structurally rigid mesogen seems to be the main criterion for LC behavior such as linearly extended benzene rings. The descriptor, fr_unbrch_alkane, was still important in any blending shown in Fig. 8 which represents the fraction of unbranched alkane. If there are some branching alkane in wing groups, it may cause the disruption of molecule packing and destabilize liquid crystals. Therefore, both size and branching of wings are important to LC predictions.

## Conclusion

The present study demonstrated that “blending” can boost the predictability and interpretability of traditional trustworthy models. The blending methods were compared regarding their ability with four different DT-based ensemble learning methods (RF, ExtraTrees, AdaBoost, and GBM) to build predictive models, for regression and classification tasks. For regression tasks of fluorescence dataset, the obtained results showed that the blending with the QC descriptors produced a model of good predictability and interpretability with good agreement with the experimental facts of fluorescence wavelengths. For classification of liquid crystal behavior, blending was also observed to exhibit better predictive performance and provided the insight into liquid crystal behaviors. Increased accuracy of the blending method is an indication that the related phenomena in the data were well-modeled. Although DT-based ensemble learning models were powerful enough to predict properties accurately, different DT-based ensemble learning models provided inconsistent predictions for the same compounds. Thus, blending methods solved the inconsistency of level-0 model predictions as long as over two level-models had correct predictions. As well as a comparison of the feature importance of the DT-based ensemble learning models and blending, the blending led to better performance and a unification interpretation of a trained model by summarizing individual predictions. The QSPR approach is a promising tool which provides quick and cost-effective for the prediction of properties of target compounds. Trust of QSPR approach can be further enhanced by interpretation when blending complements each trustworthy model in ways that conform to human knowledge and expectations.

## Supplementary information

**Additional file 1.** The supplementary material containing detail descriptor calculations, hyper-parameters of models, and feature importance outputs.

**Additional file 2.** The SDF file containing molecule structures of the fluorescence dataset.

**Additional file 3.** The training set of fluorescence with descriptors.

**Additional file 4.** The test set of fluorescence with descriptors.

**Additional file 5.** The source code used to build the prediction models.

## Data Availability

The datasets analyzed during the current study are available in the Fluorescence.org repository [http://www.fluorophores.tugraz.at/], and the commercial database, LiqCryst [http://liqcryst.chemie.uni-hamburg.de/en/program.php]. Additional file 1 is the supplementary material containing detail descriptor calculations, hyper-parameters of models, and feature importance outputs. Additional file 2 is the molecule structures of the fluorescence dataset as an SDF file. Additional files 3 and 4 is the training set and test set of fluorescence with descriptors that were used. Additional file 5 is the code used to build the models.

## References

[1] Mitchell JBO (2014). Machine learning methods in chemoinformatics. Wiley Interdiscip Rev Comput Mol Sci.

[2] Katritzky AR, Lobanov VS, Karelson M (1995). QSPR: the correlation and quantitative prediction of chemical and physical properties from structure. Chem Soc Rev.

[3] Hansch C, Maloney PP, Fujita T, Muir RM (1962). Correlation of biological activity of phenoxyacetic acids with Hammett substituent constants and partition coefficients. Nature.

[4] Breiman L, Friedman J, Stone CJ, Olshen RA (1984). Classification and regression trees.

[5] Goh ATC (1995). Back-propagation neural networks for modeling complex systems. Artif Intell Eng.

[6] Cortes C, Vapnik V (1995). Support-vector networks. Mach Learn.

[7] Kim B, Khanna R, Koyejo OO (2016) Examples are not enough, learn to criticize! criticism for interpretability. In: Advances in neural information processing systems. pp 2280–2288

[8] Lakkaraju H, Bach SH, Leskovec J (2016) Interpretable decision sets: A joint framework for description and prediction. In: Proceedings of the 22nd ACM SIGKDD international conference on knowledge discovery and data mining. pp 1675–1684 10.1145/2939672.2939874 PMC5108651 27853627

[9] Breiman L (2001). Random forests. Mach Learn.

[10] Strobl C, Boulesteix A-L, Kneib T (2008). Conditional variable importance for random forests. BMC Bioinform.

[11] Svetnik V, Liaw A, Tong C, Wang T (2004) Application of Breiman’s random forest to modeling structure–activity relationships of pharmaceutical molecules BT. In: Roli F, Kittler J, Windeatt T (eds) Multiple classifier systems: 5th international workshop, MCS 2004, Cagliari, Italy, June 9–11, 2004. Proceedings. Springer Berlin Heidelberg, Berlin, pp 334–343

[12] Teixeira AL, Leal JP, Falcao AO (2013). Random forests for feature selection in QSPR models—an application for predicting standard enthalpy of formation of hydrocarbons. J Cheminform.

[13] Guha R, Jurs PC (2004). Development of linear, ensemble, and nonlinear models for the prediction and interpretation of the biological activity of a set of PDGFR inhibitors. J Chem Inf Comput Sci.

[14] Polishchuk PG, Muratov EN, Artemenko AG (2009). Application of random forest approach to QSAR prediction of aquatic toxicity. J Chem Inf Model.

[15] Marchese Robinson RL, Palczewska A, Palczewski J, Kidley N (2017). Comparison of the predictive performance and interpretability of random forest and linear models on benchmark data sets. J Chem Inf Model.

[16] Breiman L (1996). Bagging predictors. Mach Learn.

[17] Freund Y, Schapire R, Abe N (1999). A short introduction to boosting. J Jpn Soc Artif Intell.

[18] Zhu H, Tropsha A, Fourches D (2008). Combinatorial QSAR modeling of chemical toxicants tested against *Tetrahymena pyriformis*. J Chem Inf Model.

[19] Wolpert DH (1992). Stacked generalization. Neural Netw.

[20] Bennett J, Lanning S et al (2007) The netflix prize. In: Proceedings of KDD cup and workshop. p 35

[21] fluorophores.org. http://www.fluorophores.tugraz.at/. Accessed 1 May 2007

[22] Weber G, Farris FJ (1979). Synthesis and spectral properties of a hydrophobic fluorescent probe: 6-propionyl-2-(dimethylamino)naphthalene. Biochemistry.

[23] Kucherak OA, Didier P, Mély Y, Klymchenko AS (2010). Fluorene analogues of prodan with superior fluorescence brightness and solvatochromism. J Phys Chem Lett.

[24] Lu Z, Lord SJ, Wang H (2006). Long-wavelength analogue of PRODAN: synthesis and properties of anthradan, a fluorophore with a 2,6-donor–acceptor anthracene structure. J Org Chem.

[25] Vill V (2005). LiqCryst 4.6 database.

[26] Opitz D, Maclin R (1999). Popular ensemble methods: an empirical study. J Artif Intell Res.

[27] Polikar R (2006). Ensemble based systems in decision making. IEEE Circuits Syst Mag.

[28] Rokach L (2010). Ensemble-based classifiers. Artif Intell Rev.

[29] Geurts P, Ernst D, Wehenkel L (2006). Extremely randomized trees. Mach Learn.

[30] Breiman L (1997) Arcing the edge

[31] Friedman JH (2016) Greedy function approximation: a gradient boosting machine. https://statweb.stanford.edu/~jhf/ftp/trebst.pdf

[32] Friedman JH (2002). Stochastic gradient boosting. Comput Stat Data Anal.

[33] Breiman L (1996). Stacked regressions. Mach Learn.

[34] Muratov EN, Artemenko AG, Varlamova EV (2010). Per aspera ad astra: application of simplex QSAR approach in antiviral research. Future Med Chem.

[35] Raccuglia P, Elbert KC, Adler PDF (2016). Machine-learning-assisted materials discovery using failed experiments. Nature.

[36] Kode-Chemoinformatics (2016) Dragon version 7.0.4

[37] Frisch MJ, Trucks GW, Schlegel HB, et al (2016) Gaussian 09 Revision A.02

[38] RDKit. http://rdkit.org/. Accessed 1 Apr 2017

[39] Becke AD (1993). A new mixing of Hartree–Fock and local density-functional theories. J Chem Phys.

[40] Chen C-H, Tanaka K, Funatsu K (2018). Random forest approach to QSPR study of fluorescence properties combining quantum chemical descriptors and solvent conditions. J Fluoresc.

[41] Marini A, Muñoz-Losa A, Biancardi A, Mennucci B (2010). What is solvatochromism?. J Phys Chem B.

[42] Chen C-H, Tanaka K, Funatsu K (2018). Random forest model with combined features: a practical approach to predict liquid-crystalline property. Mol Inform.

[43] Pedregosa F, Varoquaux G, Gramfort A (2011). Scikit-learn: machine learning in Python. J Mach Learn Res.

[44] Sheppard SE, Newsome PT (1942). The effect of solvents on the absorption spectra of dyes. II. Some dyes other than cyanines. J Am Chem Soc.

[45] Gray GW (1962). Molecular structure and the properties of liquid crystals.

[46] Priestly E (2012). Introduction to liquid crystals.

