# Supplementary material for: Comparison and improvement of the predictability and interpretability with ensemble learning models in QSPR applications
Source: J Cheminform. 2020 Mar 30;12:19. doi: 10.1186/s13321-020-0417-9 (PMC7106596; doi:10.1186/s13321-020-0417-9)
Supplement: Supplementary file 1 — Additional file 1. The supplementary material containing detail descriptor calculations, hyper-parameters of models, and feature importance outputs. [file 13321_2020_417_MOESM1_ESM.pdf]

Additional File

Comparison and Improvement of the Predictability and Interpretability with Ensemble Learning Models in QSPR Applications

Chia-Hsiu CHEN, Kenichi TANAKA, Masaaki KOTERA and Kimito FUNATSU*

Department of Chemical System Engineering, The University of Tokyo, 7-3-1 Hongo, Bunkyo-ku, Tokyo 113-8656, Japan

Fax:+81 35841 7771

Tel:+81 35841 7751

E-mail: funatsu@chemsys.t.u-tokyo.ac.jp

Table of Content

Section A: Descriptor calculation and selection of fluorescence dataset 3
Selection of training and test data 3
Calculation of quantum chemical (QC) descriptors 3
Descriptor selection of fluorescence 4

Section B: Descriptor selection of liquid crystal dataset 6
Separation of mesogen and wings 6
Descriptor calculation and selection of liquid crystal 6

Section C: Additional information of Case study1 8
The hyper-parameters of models in Case study 1 8
Figures of experimental values versus calculated values in Case study 1 8

Section D: Additional information of Case study 2 12
The hyper-parameters of models in Case study 2 12
Bar charts of feature importance in Case study 2 12

Reference 16

Section A: Descriptor calculation and selection of fluorescence dataset

Selection of training and test data

A complete chromophore derivative list of the dye types is given in Table S1. Two-dimensional (2D) structures were generated in a standardizer software that canonized structures, added hydrogens and performed aromatic form conversions [1]. 3D structures were optimized, and the geometries of the minimum energy conformations were obtained using the MMFF94 optimization routine the MMFF94 force field with Knime nodes[2].

Table S1. Chromophore derivatives (dye type) and number of dyes in data set.

Dye type	Number of dyes	Dye type	Number of dyes
Acridine	10	Luminogren	2
Anthracene	9	Naphthalene	8
Benzene	20	Perylene	13
Benzothiazole	3	Phenoxazine	11
Benzoxadiazole	4	Phenyloxazole	8
Benzoxazole	13	Porphyrine	10
BODIPY	16	Pyrene	12
Coumarin	50	Quinoline	2
Cyanine	123	Xanthene	79
Fluorene	2	Others	18

Calculation of quantum chemical (QC) descriptors

25 quantum mechanical properties calculated by Gaussian 09 software. The geometry optimization and molecular descriptor calculations were performed using Gaussian 09.[3] The geometries of the molecules were optimized with the B3LYP density functional method [4], using the 6–31G* basis set, and were followed by frequency calculations to verify true energy minima. The calculated quantum chemical (QC) descriptors include:

1. 2 atomic force descriptors: maximum force on molecules (FMAX), root mean square force (FRMS),
2. 4 energy descriptors: highest Occupied Molecular Orbital (HOMO), Lowest Unoccupied Molecular Orbital (LUMO), HOMO-LUMO gap, thermal energy (TE),
3. 6 charge information descriptors: minimum of negative charge (MNQ) and positive charge (MPQ), sum of negative charge (SNQ) and positive charge (SPQ), average of negative charge (ANQ) and positive charge (APQ),
4. 13 polar related descriptors: dipole moment (DP), exact polarizability, (EP(xx), EP(xy), EP(yy), EP(xz), EP(yz), EP(zz)) and approximate polarizability (AP(xx), AP(xy), AP(yy), AP(xz), AP(yz), AP(zz)).

Descriptor selection of fluorescence

In order to development of robust, predictive and interpretable models on the basis of RF, it is essential to select appropriate descriptors to construct models. We compare two models of fluorescence wavelength (λem) prediction using RF with different descriptors:

(1) RF(RDKit) model with RDKit descriptors, QC descriptors, and solvent (196 descriptors),

(2) RF model with Dragon 7 descriptors, QC descriptors, and solvent (2,169 descriptors),

The results of the three models are shown in Table S2, Figure S2, and Figure S3. The prediction result of RF is much better than the RF(RDKit) result. It might be a good suggestion that Dragon 7 descriptor is more suitable for fluorescence prediction.

Table S2. The coefficient of determination and root mean square error value for the different models.

	Training dataset		Test dataset	
	R^2	RMSE (nm)	R^2	RMSE (nm)
RF(RDKit)	0.958	24.23	0.859	41.79
RF	0.966	22.25	0.904	34.42

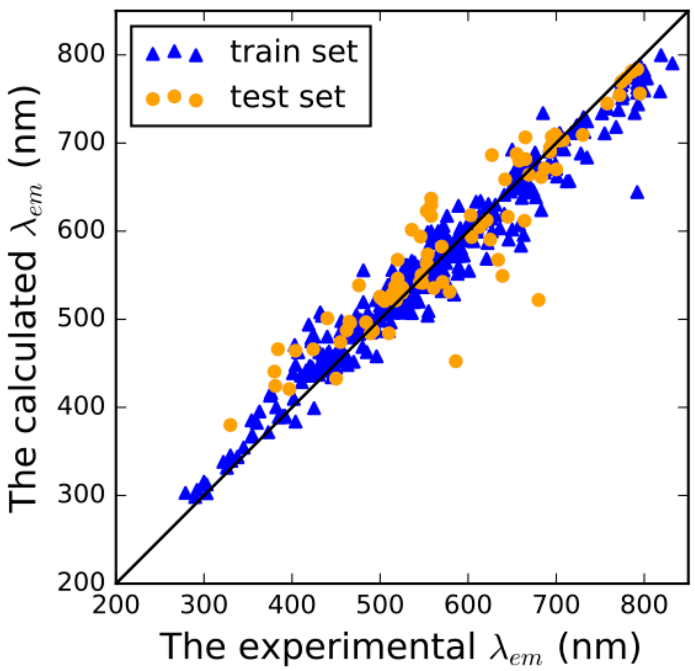

Fig. S1 Experimental values versus calculated values of λ_{em} by RF(RDKit).

Section B: Descriptor selection of liquid crystal dataset

Separation of mesogen and wings

Most LCs are combinations of rigid and flexible chains induce structural alignment and fluidity between liquid crystal moieties. Figure S1 depicts an example of LC structures. The rigid moieties, so called "mesogen", have distinctive shapes such as such as cyclohexane, benzene, and biphenol. The flexible segments (wings) provide mesogens with mobility such as alkyl chains. The mesogen types including different side chains and the flexibility of wings are important factors to LC behaviors.
We construct a program to extract LC's mesogens and wings by RDKit. The information of mesogens and wings were storage for subsequent RDkit descriptor calculation.

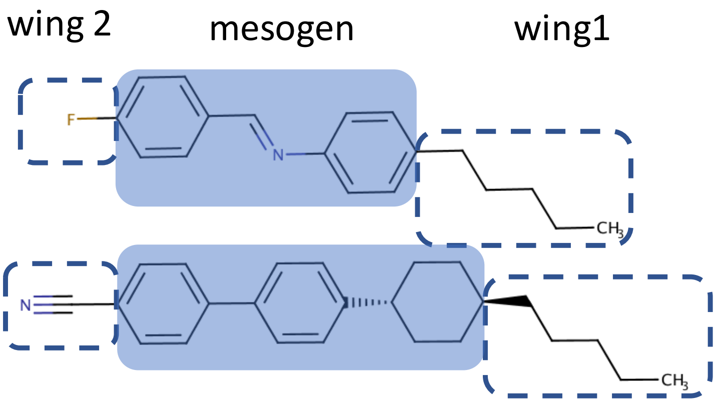

Figure S2. Structure separation of rod-like LCs.

Descriptor calculation and selection of liquid crystal

A critical part is coming up with a good set of features for both prediction and model interpretation. First, we compare the results of two RF models using 3,663 Dragon 7 descriptors and 168 RDkit descriptors. Then, we tried to separate compounds in two parts, mesogen and wing and calculate the molecular descriptors of each fragment. Table S2 shows the number of descriptors we used to construct RF classifiers. To improve interpretability, we removed all topological descriptors in RDKit_separate2 model because topological descriptors have less intuitive physical

explanations. From the results in Table S3, though, Dragon 7 has a large variety of descriptors, 168 RDkit descriptors are enough for training a RF model of LC classification. The separation of LC has slight improvement of the classification results. The remove of topological descriptors in RDKit_separate2 model did not reduce the predictability compared to RDKit_separate model. Therefore, we used 250 descriptors of RDKit_separate2 to construct models in this research.

Table S2. Descriptors for RF classifier training

Model	Dragon 7	RDKit	RDKit_separate	RDKit_separate2
Number of descriptors	3,663	168	168 (raw structure)	84 (raw structure)
			155 (mesogen)	72 (mesogen)
			122 (wings)	46 (wing1)
			125 (wing2)	48 (wing2)

Table S3. Performance metrics values for the different classifiers and corresponding confusion tables.

	Training set				Test set						
	Acc (%)	Pr (%)	r (%)	F1 (%)	Acc (%)	Pr (%)	r (%)	F1 (%)	Actual class	Predicted class LC	Predicted class NLC
Dragon 7	99.7	99.8	99.8	99.8	87.6	90.6	93.5	92.0	LC	673	47
									NLC	70	157
RDKit	99.7	99.9	99.8	99.8	87.4	90.3	93.5	91.9	LC	673	47
									NLC	71	156
RDKit separate	99.8	99.9	99.7	99.8	88.7	92.1	93.1	92.6	LC	670	50
									NLC	57	170
RDKit separate2	99.3	99.5	99.6	99.5	88.3	91.6	93.3	92.4	LC	672	48
									NLC	62	165

Section C: Additional information of Case study1

The hyper-parameters of models in Case study 1

RF: n_estimators=110, min_samples_split = 5, min_samples_leaf= 1

ExtraTrees: n_estimators=110, min_samples_split = 8, min_samples_leaf= 1

AdaBoost: base_estimator=DecisionTreeRegressor(max_depth=6), n_estimators=100, learning_rate= 1, loss='exponential'

GBM: n_estimators=100, max_depth=6, learning_rate=0.1

Level-1 GBM in any blending: n_estimators=10, max_depth=8, learning_rate=0.1

Figures of experimental values versus calculated values in Case study 1

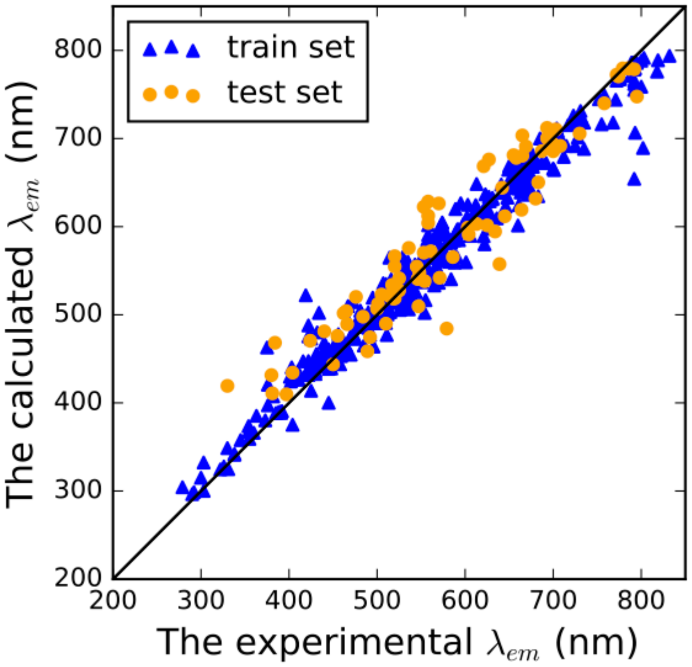

Fig. S3 Experimental values versus calculated values of λ_{em} by RF.

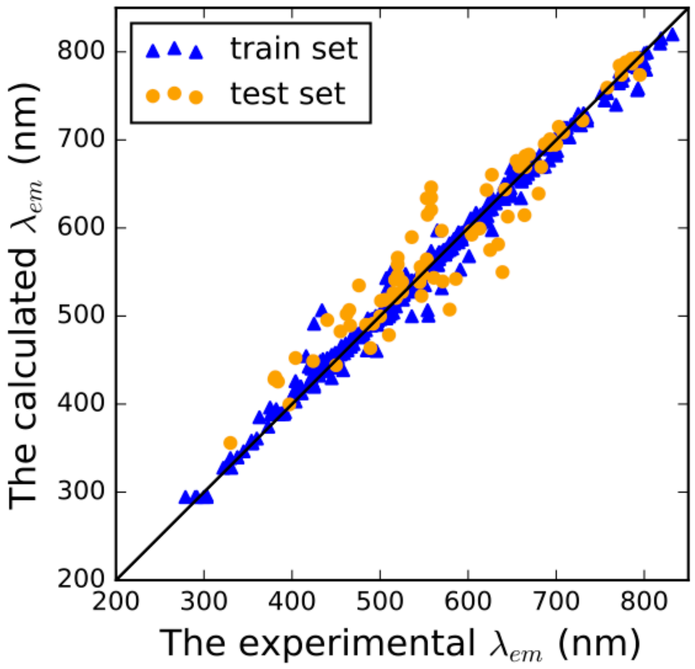

Fig. S4 Experimental values versus calculated values of λ_{em} by ExtraTrees.

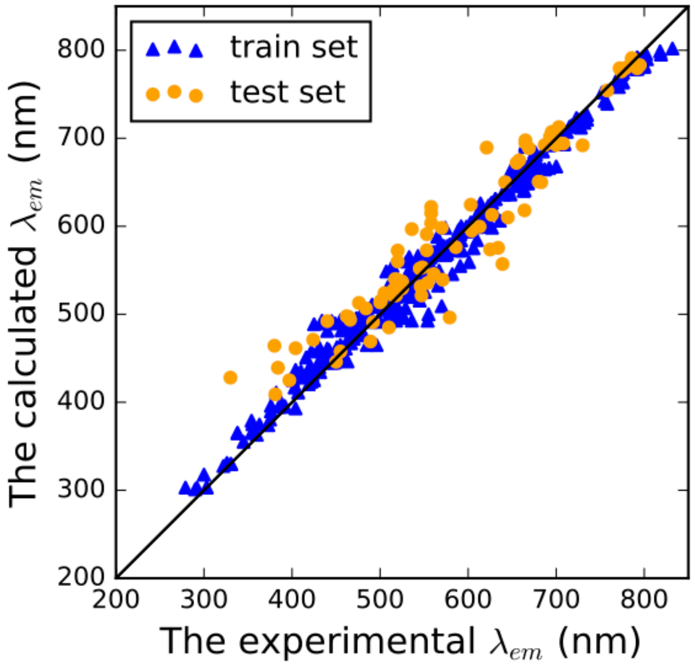

Fig. S5 Experimental values versus calculated values of λ_{em} by AdaBoost.

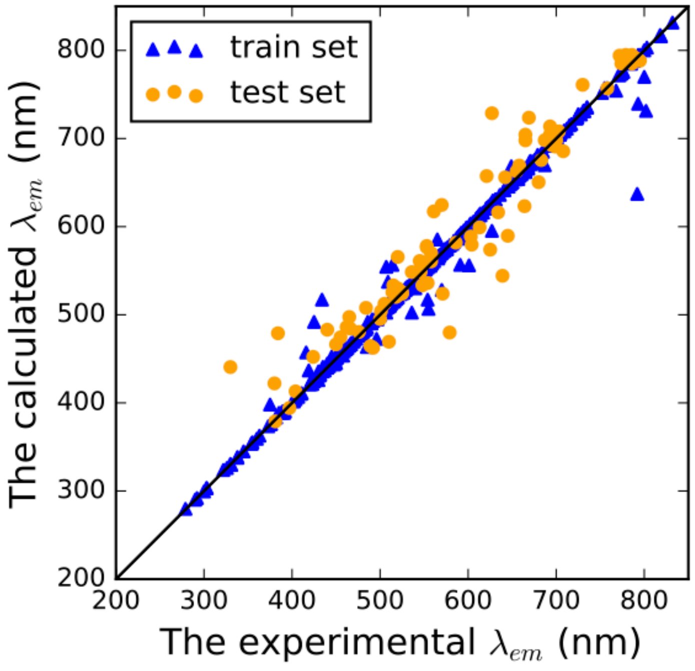

Fig. S6 Experimental values versus calculated values of λ_{em} by GBM.

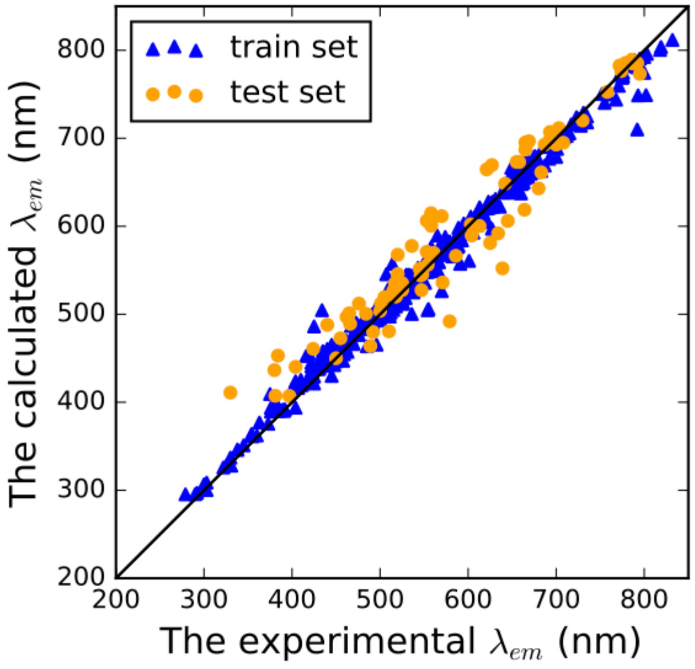

Fig. S7 Experimental values versus calculated values of λ_{em} by uniform blending.

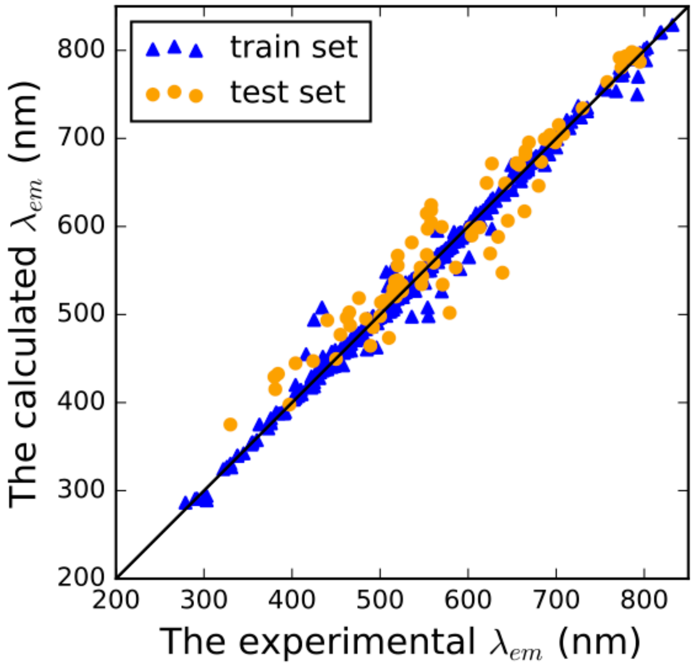

Fig. S8 Experimental values versus calculated values of λ_{em} by linear blending.

Section D: Additional information of Case study 2

The hyper-parameters of models in Case study 2

The hyper-parameters did not affect the mode training in LC prediction. Thus, we only coordinate n_estimators and max_depth.

RF:	n_estimators=400
ExtraTrees:	n_estimators=400
AdaBoost:	base_estimator=DecisionTreeRegressor(max_depth=5),<br>n_estimators=400, learning_rate= 0.1
GBM:	n_estimators=400, max_depth=5, learning_rate=0.1

Level-1 GBM in any blending: n_estimators=10, max_depth=4, learning_rate=0.2

Bar charts of feature importance in Case study 2

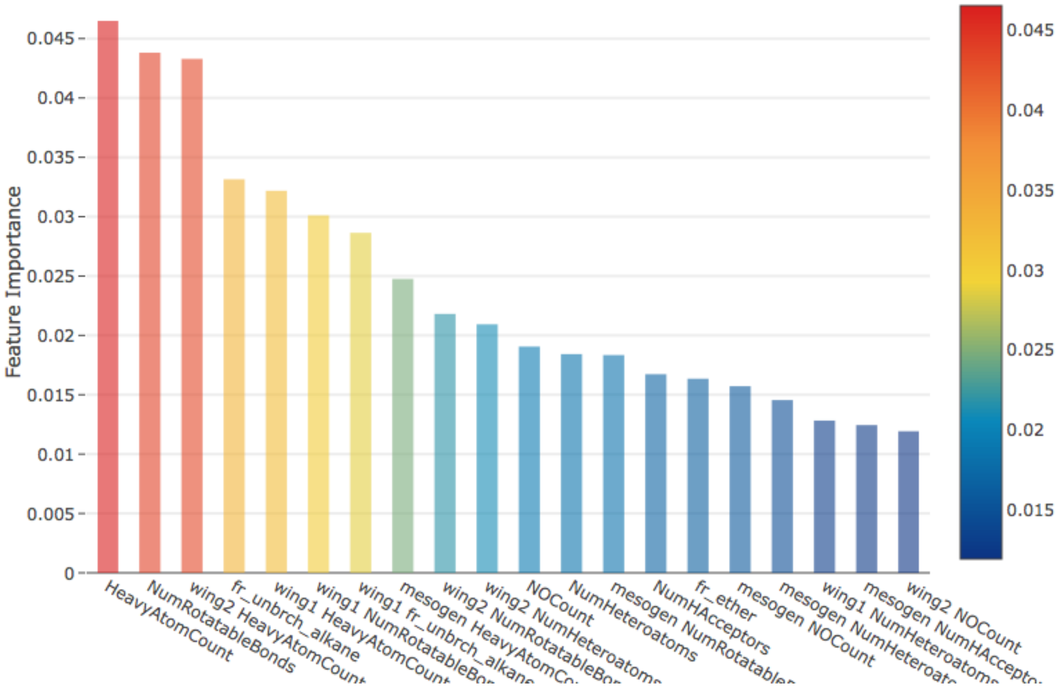

Fig. S9 Bar chart of top 20 important descriptors selected by RF.

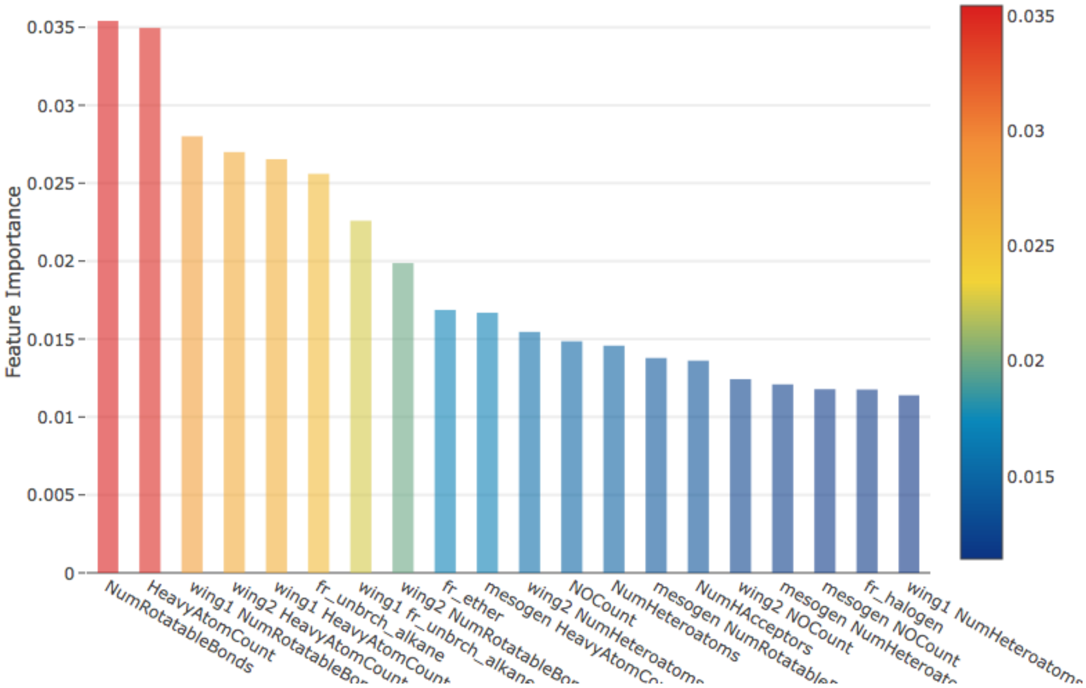

Fig. S10 Bar chart of top 20 important descriptors selected by ExtraTrees.

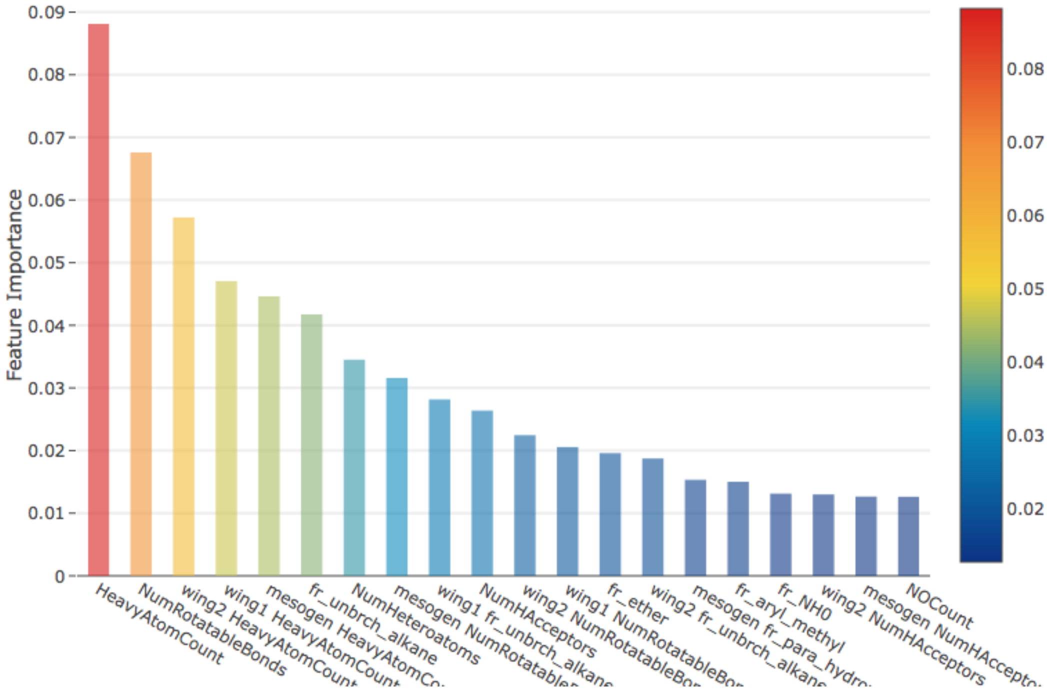

Fig. S11 Bar chart of top 20 important descriptors selected by AdaBoost.

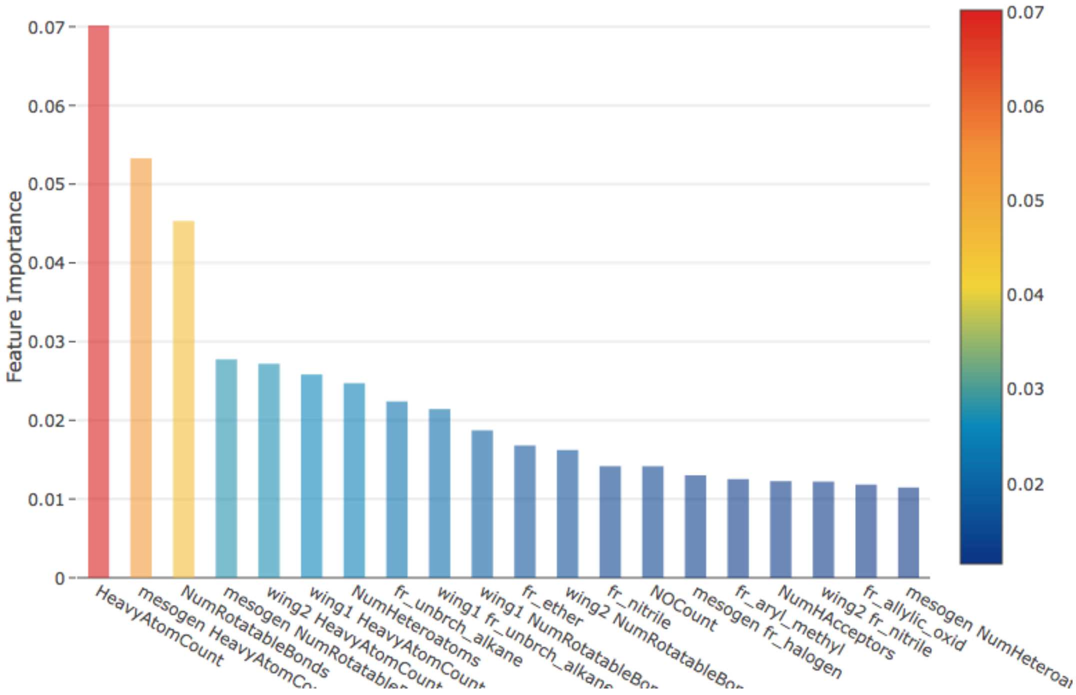

Fig. S12 Bar chart of top 20 important descriptors selected by GBM.

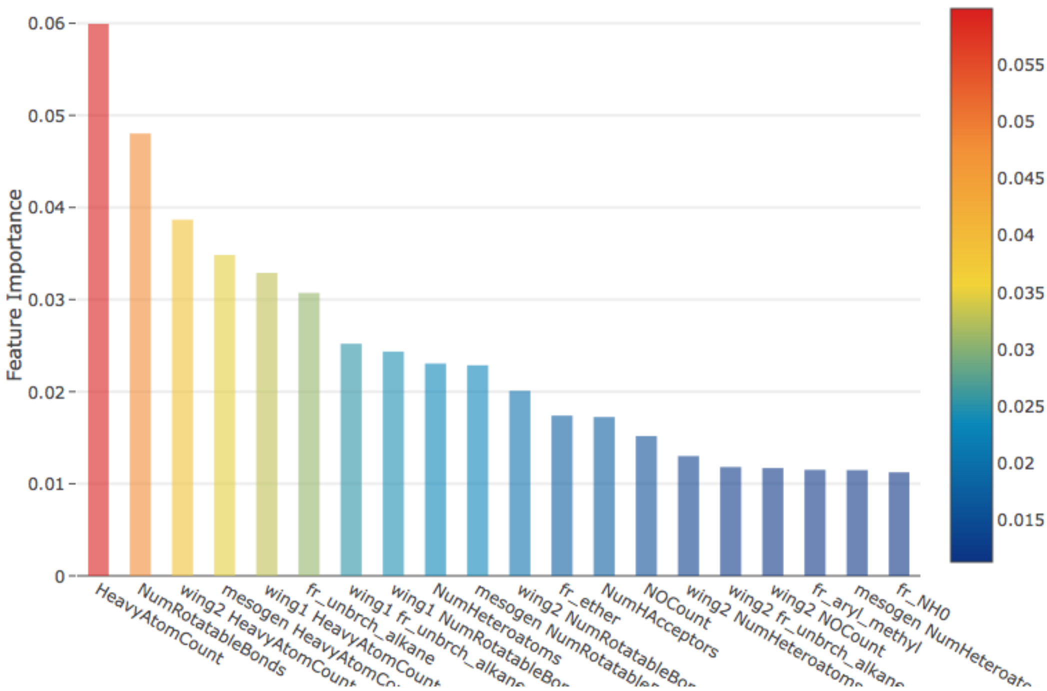

Fig. S13 Bar chart of top 20 important descriptors selected by uniform blending.

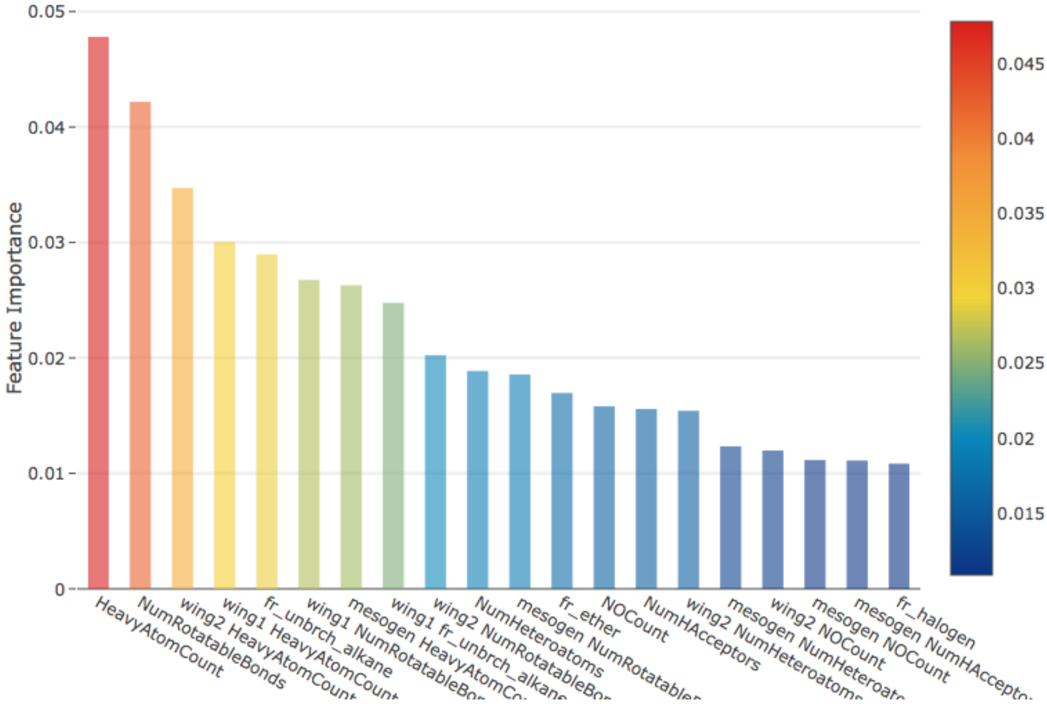

Fig. S14 Bar chart of top 20 important descriptors selected by linear blending.

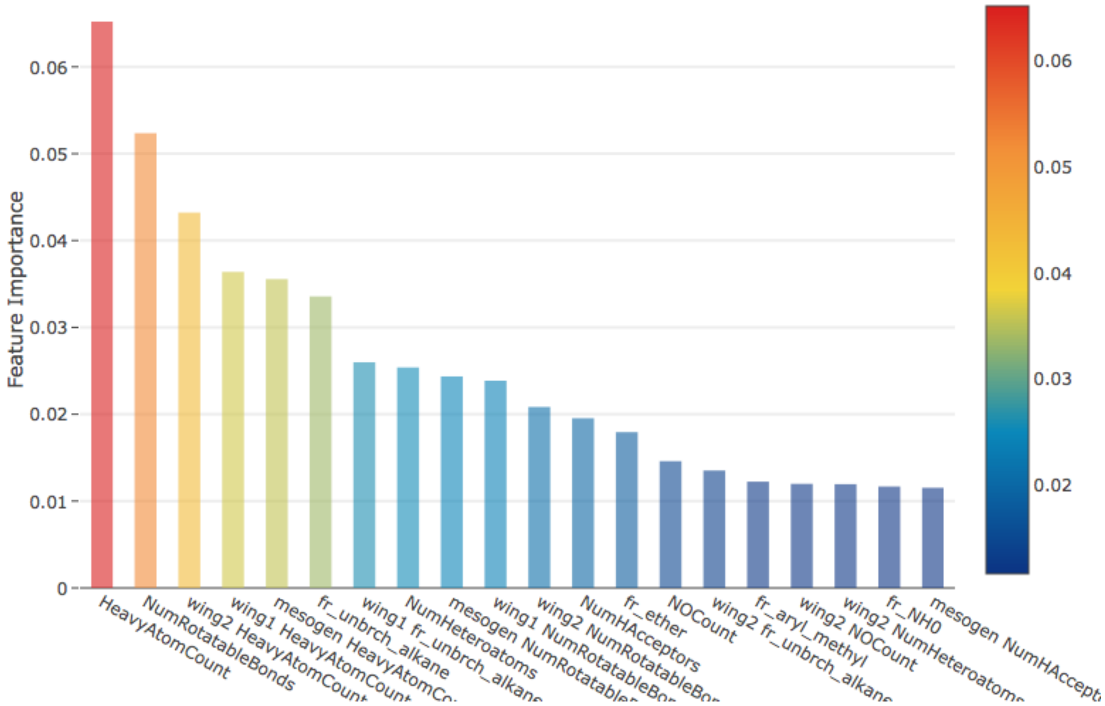

Fig. S15 Bar chart of top 20 important descriptors selected by any blending.

Reference

[1] ChemAxon (2017) Marvin 17.28.0

[2] Berthold MR, Cebron N, Dill F, et al (2009) KNIME - the Konstanz Information Miner: Version 2.0 and Beyond. SIGKDD Explor Newsl 11:26–31 . doi: 10.1145/1656274.1656280

[3] Frisch MJ, Trucks GW, Schlegel HB, et al (2016) Gaussian 09 Revision A.02

[4] Becke AD (1993) A new mixing of Hartree–Fock and local density-functional theories. J Chem Phys 98:1372–1377 . doi: 10.1063/1.464304
